# Amyloid beta accumulations and enhanced neuronal differentiation in cerebral organoids of Dutch-type cerebral amyloid angiopathy patients

**DOI:** 10.3389/fnagi.2022.1048584

**Published:** 2023-01-17

**Authors:** Elena Daoutsali, Barry A. Pepers, Stavros Stamatakis, Linda M. van der Graaf, Gisela M. Terwindt, David A. Parfitt, Ronald A. M. Buijsen, Willeke M. C. van Roon-Mom

**Affiliations:** ^1^Department of Human Genetics, Leiden University Medical Center, Leiden, Netherlands; ^2^Department of Neurology, Leiden University Medical Center, Leiden, Netherlands

**Keywords:** *in vitro* 3D disease modeling, cerebral organoids, Dutch-type cerebral amyloid angiopathy, Aβ accumulations, astrocytes

## Abstract

**Introduction:**

ADutch-type cerebral amyloid angiopathy (D-CAA) is a hereditary brain disorder caused by a point mutation in the amyloid precursor protein (APP) gene. The mutation is located within the amyloid beta (Aβ) domain of APP and leads to Aβ peptide accumulation in and around the cerebral vasculature. There lack of disease models to study the cellular and molecular pathological mechanisms of D-CAA together with the absence of a disease phenotype in vitro in overexpression cell models, as well as the limited availability of D-CAA animal models indicates the need for a D-CAA patient-derived model.

**Methods:**

We generated cerebral organoids from four D-CAA patients and four controls, cultured them up to 110 days and performed immunofluorescent and targeted gene expression analyses at two time points (D52 and D110).

**Results:**

D-CAA cerebral organoids exhibited Aβ accumulations, showed enhanced neuronal and astrocytic gene expression and TGFβ pathway de-regulation.

**Conclusions:**

These results illustrate the potential of cerebral organoids as in vitro disease model of D-CAA that can be used to understand disease mechanisms of D-CAA and can serve as therapeutic intervention platform for various Aβ-related disorders.

## Introduction

Dutch-type cerebral amyloid angiopathy (D-CAA) is a rare hereditary brain disease caused by a point mutation in the amyloid precursor protein (*APP*) gene [NM_000484.3(APP):c.2077G > C]. It occurs mainly in families from two coastal villages in the Netherlands (Bornebroek et al., 1996; Kamp et al., 2014) as well as in families in Southwest Australia (Panegyres et al., 2005). The mutation results in a glutamic acid to glutamine amino acid substitution (NP_000475.1:p.Glu693Gln) that lies within the amyloid beta (Aβ) domain of APP. Αβ peptides of varying lengths are derived from the differential amyloidogenic proteolytic processing of APP by β-and γ-secretases. In D-CAA the predominant toxic Aβ peptide is 40 amino acids long (Aβ40). The amino acid substitution from a charged residue (Glu) to an uncharged residue (Gln) results in accelerated oligomerization and fibrillization of Aβ as well as an impaired Aβ transport and clearance across the cerebral vasculature leading to its accumulation around cerebral leptomeningeal arteries and cortical arterioles causing degeneration of vascular smooth muscle cells. This will finally lead to intracerebral hemorrhage (ICH) between the ages of 40 and 65 (Bornebroek et al., 1997; Baumketner et al., 2008; Kim and Hecht, 2008; Ghiso et al., 2014). Clinically, D-CAA is characterized by hemorrhagic strokes, infarcts, vascular cognitive impairment and dementia (Bornebroek et al., 1997).

Hereditary brain diseases with a known genetic cause are helpful to design and generate cell and animal models which will lead to advancements in understanding fundamental disease mechanisms of central nervous system disorders and will serve as platforms for the development of therapeutics (Chesselet and Carmichael, 2012). However, currently there is a low success rate of translating novel disease modifying therapeutic approaches into the clinic for CAA. This may be explained in part by lack of disease etiology in disease models, the differences between humans and animal models as organisms, as well as the lack of appropriate patient-derived models. For D-CAA there are two transgenic animal models available; a mouse model overexpressing the human Dutch-APP (E693Q) that develops an Aβ phenotype at 20–22 months of age (Herzig et al., 2004) and a rat model carrying human APP containing the Swedish, Dutch and Iowa APP mutations that shows an early accumulation of cerebral microvascular Aβ fibrils and behavioral deficits at 3 months of age (Davis et al., 2018). The ability to reprogram patient-derived human somatic cells to induced pluripotent stem cells (iPSCs) has enabled new ways of disease modeling (Takahashi et al., 2007). Recently we generated and fully characterized hiPSCs from a D-CAA patient (Daoutsali et al., 2019). Human iPSCs can be differentiated towards brain specific cell-types, such as neurons, astrocytes and microglia (Shi et al., 2012; Speicher et al., 2019). However, the brain is a three-dimensional complex organ, where multiple cell types interact to form different brain regions. Lancaster et al. (2013) introduced for the first time the generation and use of cerebral organoids to model brain disorders. Cerebral organoids have since been used to model multiple neurodegenerative brain diseases, including Alzheimer’s disease (AD), frontotemporal dementia (FTD), familial and sporadic Parkinson’s disease, as well as psychiatric disorders like schizophrenia and bipolar disorder (Son et al., 2017; Kim et al., 2019; Nakamura et al., 2019; Papaspyropoulos et al., 2020; Sawada et al., 2020). Recently it was also shown that organoids have the potential to be used as a drug screening platform (Raja et al., 2016; Groveman et al., 2021; Park et al., 2021). However, these 3D models also have limitations when modeling neurodegenerative or neurovascular diseases. iPSC-induced cerebral organoids are more comparable to prenatal brain than adult brain and their lack of vascularization leads to loss of an important part of the brain physiology, the blood–brain barrier, which is important in the pathology of D-CAA (Di Lullo and Kriegstein, 2017; Huch et al., 2017). Therefore, cerebral organoid models are an additional human model, besides animal models, that can be used to identify molecular mechanisms of this vascular brain disease and may serve as a platform for therapeutic interventions.

In this study, we wanted to examine if there are changes in D-CAA cerebral organoids caused by the expression of the mutant APP protein. In a previous study, our group showed that there is no higher production of the Aβ40 and Aβ42 peptide in D-CAA neuronally differentiated iPSCs (Daoutsali et al., 2021). That is in contrast with cell and organoid models of AD, where presenilin and APP mutations, like the London and Swedish, as well as Down syndrome chromosome 21 trisomy (3 copies of APP) lead to an increased expression of the Aβ peptide (Choi et al., 2014; Gonzalez et al., 2018). Therefore, our current hypothesis is that in D-CAA there is normal production of Aβ, but the Dutch-mutation creates an Aβ peptide that is more prone to aggregation. We show that disease relevant changes can be identified in D-CAA organoids. Firstly, we compared two different cerebral organoid protocols and used the most efficient in generating organoids of the same size and morphology to grow cerebral organoids from four control and four D-CAA cell lines, including an isogenic pair created with CRISPR/Cas9 technology. D-CAA organoids exhibited Aβ accumulations after only 52 days in culture, and showed elevated expression of neuron-and astrocyte-specific genes compared to controls. Moreover, we noticed TGFβ receptor 1 upregulation at both 52 and 110 days. Cerebral organoids are therefore an important addition to existing D-CAA models and can be used to further our knowledge on D-CAA pathology, disease mechanisms and novel therapy development.

## Materials and methods

### Reprogramming of iPSC lines

The study was approved by the LUMC Medical Ethical Committee (MEC) and with patient informed consent (NL45478.058.13/P13.080). In this study four D-CAA and four control lines were used. Reprogramming of the D-CAA 3 and control 1 cell lines were previously published by our group (Daoutsali et al., 2019). Reprogramming of the remaining three control lines and quality controls were also done by our group (data not shown). Skin biopsies were obtained from two D-CAA patients. Following dissection, fibroblasts were cultured in minimum essential medium (MEM) supplemented with 15% FBS, 2 mM GlutaMAX and 1% penicillin–streptomycin (all Thermofisher) at 37°C and 5% CO_2_. Fibroblasts were expanded up to passage five and were frozen in liquid nitrogen for future use. For reprogramming of fibroblasts the ReproRNA™-OKSGM kit (StemCell technologies) was used according to manufacturer’s instructions. After 5 weeks hiPSC colonies formed and were picked manually and replated in Matrigel (Corning) coated plates in mTeSR1 (StemCell Technologies) medium. The hiPSC colonies were passaged approximately once per week and quality control tests were performed after 10 passages. hiPSCs were first checked for the expression of pluripotency markers *via* immunofluorescence. Cells were fixed in 4% paraformaldehyde (PFA) for 10 min at room temperature (RT), permeabilized with 0.1% Triton-X in PBS and probed with primary antibodies, against Oct3/4, SSEA-4 and Nanog in immunobuffer containing 1% goat serum and 0.1% Triton X-100 in PBS. To check pluripotency gene expression levels, RNA was isolated from the hiPSCs with the ReliaPrep Miniprep System (Promega) according to manufacturer’s instructions. 500 ng of RNA was used as input for cDNA synthesis with the Transcriptor first strand cDNA synthesis kit (Roche). qRT-PCR was run on a LightCycler 480 Real-Time PCR system (Roche) with SensiMix SYBR Hi-ROX Kit (Bioline) using the following program: denaturation at 95°C for 10 min, 45 cycles of denaturation at 95°C for 10s, annealing at 60°C for 30s and extension at 72°C for 20s. CT-values were normalized using the ΔΔCT-method. Primer sequences are listed in Table 1. The Global Screening Array (GSA; Illumina) was used according to standard protocols, followed by a standard analysis in GenomeStudio software (Illumina) with the GSA manifest files. GenomeStudio Finalreports was used to analyze and visualize in Nexus Discovery (BioDiscovery El Segundo). A report resolution of ~50 kb was used to analyze the data for chromosomal aberrations. To compare the patient fibroblasts with the generated hiPSCs the array final reports were selected as input for an R script. The files were transformed into smaller tables based on their SNP ID. Using statistics we determined whether the allelic calls matched, mismatched or failed. The Dutch mutation was confirmed by Sanger sequencing. Finally, we tested the cell lines for their ability to spontaneously differentiate in the 3 germ layers, endoderm, mesoderm and ectoderm. Cells were cultured in DMEM/F12 with 20% FBS for 3 weeks with media changes every other day. HiPSCs were fixated with 4% PFA and immunostained with antibodies against SOX17 (endoderm), SMA (mesoderm) and PAX6 (ectoderm).

**Table 1:** Primer sequences.

Gene	Forward primer	Reverse primer
OCT4	TGTACTCCTCGGTCCCTTTC	TCCAGGTTTTCTTTCCTAGC
NANOG	CAGTCTGGACACTGGCTGAA	CTCGCTGATTAGGCTCCAAC
SOX1	GGAATGGGAGGACAGGATTT	GGCCCGTATTAACACTCAGC
SOX2	GCTAGTCTCCAAGCGACGAA	GCAAGAAGCCTCTCCTTGAA
BCL11B (CTIP2)	TGGGGAAGTCAGTTTCTTGG	TCGTTCTGGATCTGCAACAG
PAX6	AGTGAATCAGCTCGGTGGTGTCTT	TGCAGAATTCGGGAAATGTCGCAC
FOXG1	CCAGACCAGTTACTTTTTCCC	TGAAATAATCAGACAGTCCCCC
BF1	AGGAGGGCGAGAAGAAGAAC	TGAACTCGTAGATGCCGTTG
NESTIN	CAGCTGGCGCACCTCAAGATG	AGGGAAGTTGGGCTCAGGACTGG
TUJ1	GAACCCGGAACCATGGACAG	GACCCTTGGCCCAGTTGTTG
MAP2	TGCCATCTTGGTGCCGA	CTTGACATTACCACCTCCAGGT
vGLUT1	TCAATAACAGCACGACCCAC	TCCTGGAATCTGAGTGACAATG
vGLUT2	CATGTTTTGGCTTTTGGTGTC	GGGCGAGATGGGAATAAGAT
vGAT	CAAGAAGTTCCCCATCTCCA	CGTGATGACCTCCTTGGTCT
Brachyury	AGAGCCTGCAGTACCGAGTG	ACGATCATCTCATTGGTGAGC
AIF1 (IBA1)	GGTGAGAAACGGGTGATTTG	GTGGGGAGACCCTCTCTCTC
P2RY12	GGATACATTCAAACCCTCCAG	GAGGACCTGGGTGATTTTGTAG
TMEM119	AGTCCTGTACGCCAAGGAAC	AGCAGCAACAGAAGGATGAG
GFAP	ACCAGGACCTGCTCAATGTC	ATCTCCACGGTCTTCACCAC
S100β	AGGGAGACAAGCACAAGCTGAAGA	TGTCCACAACCTCCTGCTCTTTGA
OLIG2	AGGACAAGAAGCAAATGACAG	TCCATGGCGATGTTGAGG
TGFB1	TACCTGAACCCGTGTTGCTC	GTATCGCCAGGAATTGTTGC
TGFBR1	CGTGCTGACATCTATGCAATG	TCAACTGATGGGTCAGAAGG
TGFB2	CAATGCCAACTTCTGTGCTG	ATATAAGCTCAGGACCCTGCTG
TGFBR2	CTGTGTCGAAAGCATGAAGG	AGTCAACGTCTCACACACCATC
HSPA1A	TGTGTAACCCCATCATCAGC	TCTTGGAAAGGCCCCTAATC
HSP70A6	ACCACCTACTCGGACAACC	CACGCTCAGGATGCCATTAG
DNAJB1	GAAAAGGCATTCCAGTCTGC	CCAGCCAGAAGCAAAAAGAC
AQP4 (aquaporin 4)	AGCCTGGGATGCACCATCA	TGCAATGCTGAGTCCAAAGC
ACTINB	GGATGCAGAAGGAGATCACTG	CGATCCACACGGAGTACTTG
GAPDH	GAGTCAACGGATTTGGTCGT	GACAAGCTTCCCGTTCTCAG
hHRPL22	TCGCTCACCTCCCTTTCTAA	TCACGGTGATCTTGCTCTTG
hHBMS	GCAACGGCGGAAGAAAA	CGAGGCTTTCAATGTTGCC

### CRISPR/Cas9 editing of APP gene to create D-CAA isogenic iPSC line

The online design tool http://crispr.mit.edu/ (Zhang lab, MIT) was used to design single-guide RNAs (sgRNAs) with high specificity to APP target sequences close to the desired mutation site with the least number of predicted off-targets (Table 2). Prior to nucleofection, control hiPSCs were pretreated with mTeSR1 medium supplemented with 1x CloneR (StemCell Technologies) for at least 1 h. Between 0.5-1 × 10^6^ cells were nucleofected using the Human Stem Cell Nucleofector kit 2 (Lonza) and the Amaxa Nucleofector 2b Device (program B-016), according to manufacturer’s instructions. Ribonucleoprotein (RNP) complexes were formed by combining 11.5 μg of Cas9 (Streptococcus pyogenes; Integrated DNA Technologies (IDT)) and 3 μg of sgRNA (IDT). Before electroporation, 75 pmol of ssDNA HDR donor template and 75 pmol Cas9 electroporation enhancer (IDT) was added to the Cas9 RNP solution. Cells were transferred into wells of a Matrigel-coated 24-well plate and cultured in 500 μl mTeSR1 containing 1x CloneR. Medium was changed daily and depending on cell survival rate, CloneR was omitted 24–72 h later. When hiPSCs were 80–90% confluent, the cells were washed with PBS and released with accutase (Thermofisher). Half of the detached cells were used for genomic DNA extraction, and the other half were frozen in HyClone Fetal Bovine Serum (Thermo Scientific) with 10% DMSO (Sigma) at −80°C for future sib-selection.

**Table 2:** ddPCR probe-and sgRNA sequences for the generation of the D-CAAiso hiPSC line.

Name	Sequence
APP-D-CAA ddPCR F5	CCAAATGTCCCCTGCATTTA
APP-D-CAA ddPCR R1	ACAACACCGCCCACCAT
APP-C3 (G/-SM)	FAM/TGAACCCAC/ZEN/ATCTTCTGCAAAGAACAC/IABkFQ
APP-M3 (G > C/+SM)	HEX/TGAACCGAC/ZEN/GTCTTGTGCAAAGAACAC/IABkFQ
D-CAA sgRNA1C	GTGTTCTTTGCAGAAGATGT
APP-M (G > C) (sgRNA1) HDR NT	C*TTTTTCTTAATTTGTTTTCAAGGTGTTCTTTGCACAAGACGTCGGTTCAAACAAAGGTGCAATCATTGGACTCATGG*T

Genomic DNA was extracted from the iPSCs using the ReliaPrep gDNA Tissue Miniprep System (Promega). Quantitative DNA analysis to determine the amount of single-base substitution by CRISPR editing was done by droplet digital PCR (ddPCR) using ddPCR Supermix for probes (no dUTP; Bio-Rad) and specific primers (18 μM) and dual-labeled (FAM/HEX) hydrolysis probes (5 μM) indicated in Table 2. Using 50–150 ng genomic DNA solution ddPCR was conducted with the QX200 Droplet Digital PCR System and T100 Thermal Cycler (Bio-Rad) using the following conditions: denaturation at 95°C for 10 min, 40 cycles of denaturation at 94°C for 30 s, annealing at 61°C for 60 s, and extension at 98°C for 20 s.

To obtain a pure heterozygous D-CAA mutant clone from the sib-selection screen, hiPSCs showing the highest CRISPR modification were thawed and grown in mTeSR1 containing 1x CloneR. Cells were dispersed at a low density of 1,500–2000 cells/well into Matrigel-coated 10 cm dishes and grown in mTeSR1 containing 1x CloneR. About 7–10 days later, large colonies were picked, transferred to a Matrigel-coated 48-well plate and expanded. Sanger sequencing results confirmed isolation of heterozygous clone for the D-CAA mutant. After expansion the D-CAAiso line was subjected to the hiPSC quality controls mentioned in the previous section.

### Cerebral organoid culture

Two protocols were used for the generation of cerebral organoids; the STEMdiff cerebral organoid kit (Stemcell Technologies) that is based on the publication by Lancaster et al. (2013) and the protocol described by Gabriel and Gopalakrishnan (2017), which has some minor modifications compared to the Lancaster protocol. For both protocols, cerebral organoids were generated according to the manufacturer’s or author’s instructions, respectively. In brief, the cerebral organoid culture started by plating a single cell suspension of hiPSCs in 96-well plates to generate embryoid bodies (EBs). EBs/neurospheres were embedded in matrigel droplets to support the expansion of neuroepithelium and transferred to an orbital shaker in maturation medium under maintained rotation conditions (65 rpm for a 6-well plate in the orbital shaker). The medium and time point that each medium was used are shown in Table 3. For the comparison of the two protocols cerebral organoids were collected for fixation at days 52 and 110 and were assessed using immunofluorescent analysis as described below. Finally, the STEMdiff cerebral organoid kit was selected and used according to manufacturer’s instructions to generate cerebral organoids from control and D-CAA lines. To measure the size of the organoids, brightfield images of six different organoids per cell line and time point were imported in ImageJ where the area of each organoid was reported in μm^2^_._

**Table 3:** Cerebral organoid protocol time points and media.

Day	STEMdiff kit	Gabriel and Gopalakrishnan (2017)
0	EB formation medium	Neural induction medium (NIM-Stemcell Technologies)
5	Induction medium	Matrigel embedding and neurosphere medium
7	Matrigel embedding and expansion medium	Neurosphere medium
10	Maturation medium and orbital shaker/bioreactor	Brain organoid medium and orbital shaker/bioreactor

### Immunofluorescent analysis and imaging of cerebral organoids

Three cerebral organoids of each cell line were collected at days 52 and 110 for immunofluorescent analysis. The organoids were washed with PBS and fixed in 4% PFA solution for 30 min at room RT. After fixation, the organoids were washed with PBS before incubation overnight at 4°C in a 30% sucrose solution for cryoprotection. After removing the 30% sucrose solution, the organoids were embedded in optimum cutting temperature compound, Tissue-Tek O.C.T (Sakura Finetek, United States) in Peel-A-Way embedding molds. Three organoids per cell line were placed in each embedding mold. The molds were snap-frozen in ethanol on dry-ice and were stored at-80oC until further use. For the immunofluorescent analysis the cryoprotected frozen cerebral organoids were sectioned in a cryostat, in 16 μm-thick sections that were collected on PLL-coated glass cryoslides. For the immunofluorescent stainings the cryoslides were allowed to air-dry first for 30 min at RT. The slides were washed once with PBS and then twice with 200 mM glycine in PBS to quench the PFA-induced autofluorescence. Organoid sections were blocked for non-specific antibody binding with blocking solution containing either 5% goat or horse serum and 0.1% Triton X-100 in PBS for 30 min at RT. Primary antibodies were diluted in immunobuffer solution containing either 1% goat or horse serum and 0.1% triton X-100 in PBS. Sections were incubated with primary antibodies overnight at 4°C. After primary antibody probing the slides were washed three times with PBS before secondary antibodies and DAPI diluted in immunobuffer were added on top of the sections for 2 h at RT. After secondary antibody probing the slides were washed three times with PBS. Without letting the sections dry, any excess liquid was wiped carefully off the slide and one drop of Prolong Diamond antifade mounting medium (Invitrogen) was added on top of the organoid sections together with a coverslip. The slides were left overnight at RT and were later stored at 4°C in the dark until confocal microscopy. For the immunofluorescent analysis for every cell line 3 organoids and 12 organoid sections were stained with indicated antibodies. For the overview images of the cerebral organoid sections the slide scanner Axio Scan.Z1 (Zeiss) microscope was used. For the high magnification images the confocal TCS SP8 (Leica) was used. The antibodies and dilutions used are listed in Table 4.

**Table 4:** Antibodies for immunofluorescent stainings.

Antibody name	Dilution	RRID
Oct3/4	1:200	AB_2801346
Nanog	1:200	AB_2665475
SSEA-4	1:100	AB_778073
SOX17	1:100	AB_355060
α-Smooth Muscle Actin (SMA)	1:400	AB_476701
PAX6	1:500	Stemcell Technologies, 60,094, rabbit
βTUBBIII	1:1000	Stemcell Technologies, 2G10-TB3, mouse
MAP2	1:500	AB_11006358
FOXG1	1:200	AB_732415
ZO-1	1:300	AB_10733242
CTIP2	1:500	AB_2064130
TTE	1:100	AB_2809911
GFAP	1:300	AB_10013382
IBA1	1:500	AB_839504
Y188	1:500	AB_2289606
4G8	1:500	AB_2564633
6E10	1:500	AB_2715854
AT8 (phosphorylated tau)	1:200	AB_223647
Goat anti-mouse Alexa 594	1:1000	AB_141372
Goat anti-rabbit Alexa 594	1:1000	AB_141359
Goat anti-rat Alexa 594	1:1000	AB_141374
Goat anti-rabbit Alexa 488	1:1000	AB_2576217
Goat anti-mouse Alexa 647	1:1000	AB_2633277
Donkey anti-goat Alexa 488	1:1000	AB_2762838

### RNA analysis

For RNA analysis, three organoids per cell line were collected at days 52 and 110. The organoids were washed with PBS and frozen at-20oC. For RNA isolation the ReliaPrep RNA cell miniprep system (Promega) was used according to manufacturer’s instructions with a modification in the cell lysis step. For the cell lysis, lysis buffer was added to each tube containing an organoid and triturated to disperse the cell pellet/organoid. The cell lysate was transferred to a MagNA Lyser tube (Roche) for homogenization in a bullet blender at intensity 8 for 3 min (or 3 × 1 min). After homogenization the lysates were centrifuged for 3 min at 10.000 × *g* and the supernatant was transferred into a new tube to continue with the protocol according to manufacturer’s instructions. RNA concentration was measured by Qubit Fluorometer (Invitrogen) and RNA samples were stored at-80oC. For cDNA synthesis 100 ng of total RNA from each organoid was used as template in the Transcriptor first strand cDNA synthesis kit with random hexamer primers (Roche). We validated our primer sets with RT-qPCR using a LightCycler 480 Real-Time PCR System (Roche) with SsoFast EvaGreen Supermix with low ROX (BioRad). The cycling parameters of the qPCR included an initial denaturation at 95°C for 10 s, annealing at 60°C for 30 s, and extension at 72°C for 20 s. CT-values were normalized to GAPDH using the ΔΔCT-method.

### Fluidigm RT-qPCR

For the Fluidigm platform (Fluidigm Corporation) 1.25 μl of each cDNA sample was preamplified using 1 μl of Preamp Master Mix (Fluidigm PN 100–5,580) and 0.5 μl of the primer pool. The preamplification included a denaturation step at 95°C for 2 min, followed by 15 cycles of 95°C for 15 s and 60°C for 4 min. The preamplified reactions were cleaned up with Exonuclease I (ExoI company) and after inactivation of ExoI the preamplified samples were diluted 5-fold in DNA suspension buffer (TEKnova, PN T0221). For the targeted gene expression analysis with Fluidigm, 2.5 μl of preamplified sample was mixed with 2.25 μl of 2x SsoFast EvaGreen Supermix with low ROX (BioRad) and 0.25 μl of the 20x DNA binding dye and the total volume of 5 μl was loaded into each sample inlet of the 96.96 dynamic array chip (Fluidigm Corporation). Into each assay inlet, 5 μl from an assay mix containing 2.5 μl 2x assay loading reagent, 2.25 μl 1x DNA suspension buffer and 100 μM forward and reverse primers were loaded. The 96.96 chip was placed on the NanoFlexTM 4-IFC controller for loading and mixing. After loading the chip was placed on the BioMarkTM Real-Time PCR system using a cycling program of 95°C for 10 min, followed by 40 cycles of 95°C for 15 s, 60°C for 30 s, and 72^o^c for 30 s. The raw Cq values were analyzed and obtained using the BioMark gene expression data analysis software. Samples with Cq values between 6 and 22 were used for further analysis. Four reference genes were used to correct for differences in input RNA: *ACTB*, *GAPDH*, *hRLP22*, and *hHBMS*. According to the Cq values, *ACTB* and *GAPDH* were highly expressed, whereas *hHRLP22* and *hHBMS* were lowly expressed. Normalization of highly expressed genes (Cq value ≤13) was done with the geometric mean of *ACTB* and *GAPDH*, whereas genes that were lowly expressed (Cq value ≥14) were normalized with the geometric mean of *hHRLP22* and *hHBMS*. The 96.96 array chip accommodated 42 samples in 2 different dilutions and 42 genes in duplicate (duplicate values were averaged). The primer sequences that were used are listed in Table 1.

### Statistical analysis

Statistical analyses were performed using GraphPad Prism 8.1.1. For the Fluidigm analysis the two conditions control versus D-CAA were compared first. Then for each condition changes over time were assessed. For the comparisons we used multiple t-testing with a post-hoc test (Bonferroni).

## Results

### Generation of two novel D-CAA iPSC lines

Skin biopsies were obtained from two D-CAA patients with the E693Q mutation. Following dissection, fibroblasts were cultured and reprogrammed at low passage number as previously described (Daoutsali et al., 2019). The two hiPSC lines were named D-CAA 1 and D-CAA 2 (Supplementary Figure 1). D-CAA 1 and D-CAA 2 iPSCs showed typical morphology with small and tightly packed cells, a high nucleus to cytoplasm ratio and well defined nucleoli (Supplementary Figure 1A). D-CAA iPSC lines expressed the pluripotency markers OCT3/4, Nanog and SSEA-4 as seen by immunofluorescent analysis (Supplementary Figure 1B). There was an upregulated expression of pluripotency genes *OCT4*, *NANOG*, and *SOX2 via* qPCR compared to fibroblasts (Supplementary Figure 1C). To check for copy number variants (CNV) or allelic changes and to compare the identities of the fibroblasts and derived hiPSCs a routine Global screening Array (GSA) was performed and with a resolution of ~50 kb no chromosomal aberrations were observed (Supplementary Figure 1D). The Dutch mutation was confirmed in both cell lines by Sanger sequencing (Supplementary Figure 1E). Finally, both cell lines showed spontaneous differentiation into the three germ layers endoderm, mesoderm and ectoderm as seen by immunofluorescent staining with SOX17, SMA and PAX6 antibodies, respectively (Supplementary Figure 1F). The hiPSC lines were negative in tests for mycoplasma.

### Generation of an isogenic D-CAA line

Using CRISPR/Cas9 technology on a control cell line (hereafter mentioned as control-iso), we generated an isogenic D-CAA line (D-CAAiso; APP c.2077G > C, p.E693Q). The control line had already passed successfully all quality control tests; Oct3/4, Nanog and SSEA-4 immunofluorescent stainings, *OCT4*, *NANOG*, and *SOX2* gene expression with qRT-PCR, trilineage differentiation and GSA array for chromosomal aberrations (data not shown but available with the author). The isogenic D-CAA line displayed normal iPSC morphology (Supplementary Figure 2Α) and an additional GSA screening showed that there were no chromosomal translocations. The G to C transversion was confirmed by ddPCR as well as by Sanger sequencing (Supplementary Figure 2Β). Moreover, the D-CAAiso hiPSC line successfully differentiated in the three germ layers (Supplementary Figure 2C). The results of the hiPSC quality controls showed that the D-CAAiso cell line was similar to its isogenic control line.

### Comparison of two forebrain organoid protocols

In order to minimize the variability of size and morphology of forebrain organoids, we compared two different forebrain organoid culture protocols. Organoid morphology appeared to be different on day 10 when cerebral organoids normally exhibit neuroepithelial bud formation; organoids from the Gabriel et al. protocol are missing the neuroepithelial bud formation compared to Stemcell Technologies kit (SCT) organoids (Supplementary Figure 3A). Organoid size and shape throughout the culture period showed that the organoids generated with the protocol from Gabriel et al. displayed a larger variation in size when compared to the organoids made using the SCT kit (Supplementary Figure 3B). Immunofluorescent staining with antibodies against PAX6 (an NPC-specific gene involved in the specification of forebrain neuron identity that marks neuronal precursor cells) and βTUBBIII (neurons) showed similar cortical plate structures on day 52 in both methods. In all following experiments we used the Stemcell Technologies kit for cerebral organoid culture.

### Characterization of hiPSC-derived cerebral organoids

**Figure 1:**
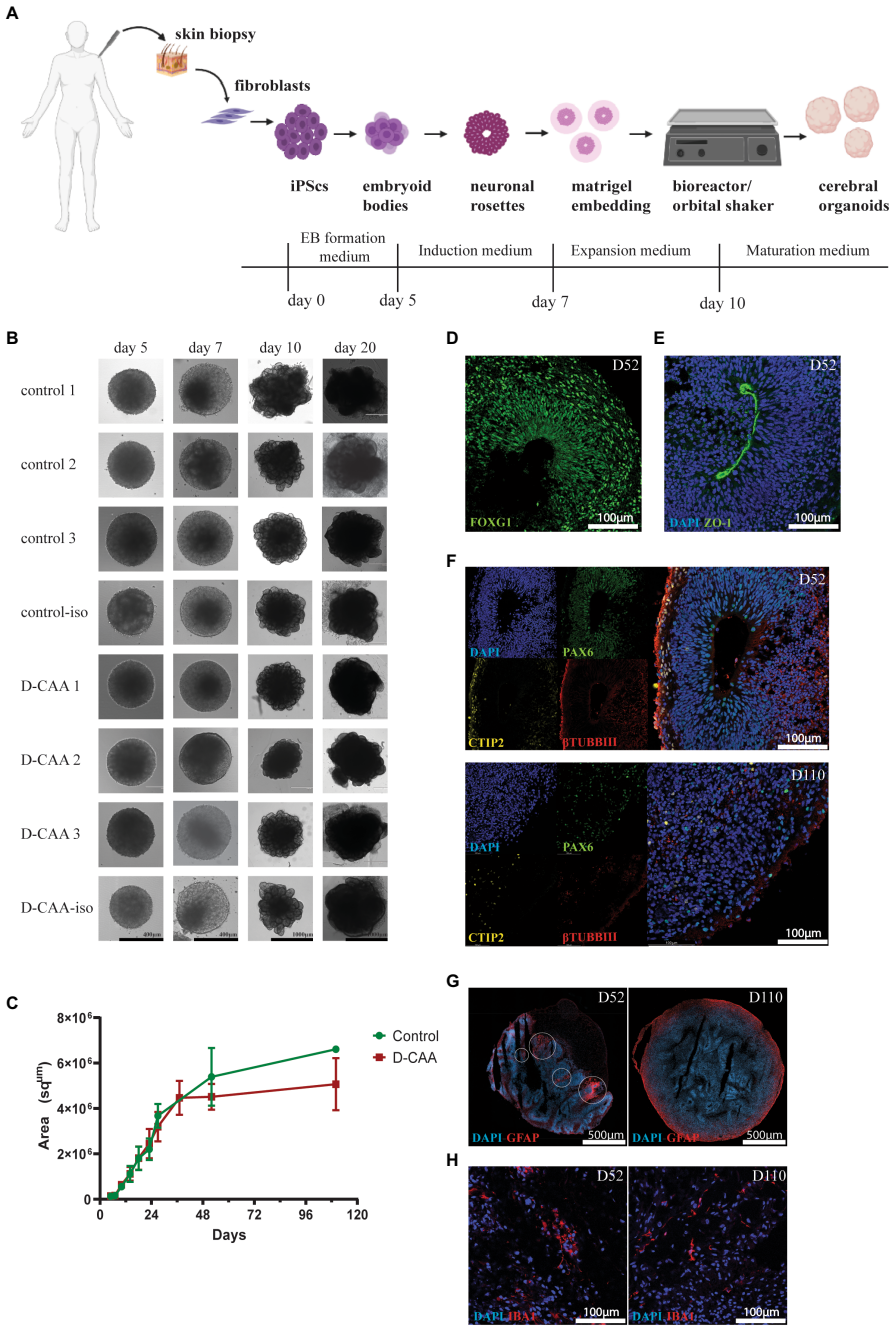
Morphological characterization of human cerebral organoids. **(A)** Schematic representation of the cerebral organoid protocol (created with BioRender.com). **(B)** Brightfield images of D-CAA and control lines. The images depict the four important steps of the cerebral organoid protocol; (Continued)FIGURE 1 (Continued)EB formation (day 5), EB induction (day 7), neuronal rosette expansion (day 10), and cerebral organoid maturation (day 20). **(C)** Size quantification of control and D-CAA organoids for the first 110 days in culture. For every time point 2–6 images for every organoid were taken. **(D)** Representative confocal image of immunofluorescent staining with an antibody against FOXG1 showing forebrain neurons in day 52 control organoids. **(E)** Representative confocal image of immunofluorescent staining with the tight junction antibody ZO-1 (green) in day 52 control organoids. Nuclei are labelled with DAPI (blue). **(F)** Representative confocal images showing the cortical layer formation using antibodies against PAX6 for neuronal precursor cells, CTIP2 for layer V neurons and βTUBIII for new-born neurons in day 52 and day 110 control organoids. **(G)** Representative image of GFAP-positive astrocytes in day 52 and day 110 control cerebral organoids. Images were made with the slide scanner Axio Scan.Z1. **(H)** Representative confocal images of IBA1 positive microglia in D52 control cerebral organoids.

For forebrain organoid generation the four main steps are: embryoid body formation, induction, expansion and maturation (Figure 1A). Brightfield images of the organoids were taken at each step for morphological observation and size measurement was done using ImageJ software. There was no difference in organoid size and morphology between the D-CAA and control organoids throughout the culture period (Figures 1B,C). For our downstream analyses we have used 2 time points; day 52 and day 110. The time points chosen were based on previous literature, where it was shown that day 52 organoids show expression of microglia among other cell types (Ormel et al., 2018) and Aβ accumulations were found after 90 and 110 days in culture (Raja et al., 2016; Gonzalez et al., 2018). Next we assessed the day 52 organoids with immunofluorescent analysis. We performed staining for the forebrain marker FOXG1 (Figure 1D) that showed typical cerebral cortical morphology. The cortical plates of the cerebral organoids exhibited tight junction structures, resembling a ventricle, with characteristic apical localization of the marker ZO-1 (Figure 1E). Previous studies on the generation of cerebral organoids have already shown the appropriate quality checks for the characterization of cortical plates (Kadoshima et al., 2013; Lancaster et al., 2017; Krefft et al., 2018; Yakoub, 2019). We accordingly analyzed the organization of these cortical regions staining for markers of radial glial cells (RGs) using the PAX6 antibody, deep-layer neurons with the CTIP2 antibody (Lancaster et al., 2013; Ballout et al., 2016; Rosebrock et al., 2022) and neurons with βTUBBIII antibody. There was a distinct expression pattern of the neuronal progenitor cell (NPC) marker PAX6 in the inner layer, the deep cortical layer marker CTIP2 in the middle and the neuronal marker βTUBBIII in the outer layer of the cortical plates (Figure 1F; Supplementary Figure 4). Even though the markers of cortical regions were present in all organoids, we notice that control organoids do not always show distinct cortical plate formation on day 52 (Supplementary Figure 4). This difference could be explained by the variability in cortical plate orientation inside the organoid; since organoid sections are used, it is highly probable that there are organoid areas were cortical plates are not present. Day 110 D-CAA and control organoids were also assessed for the aforementioned markers. Even though there was positive signal for PAX6, CTIP2 and βTUBBIII, all lines lost the typical cortical plate organization, something that is common in older organoids (Figure 1F; Supplementary Figure 4). Isogenic control and D-CAA cerebral organoids of day 52 and 110 were tested for the expression of the radial glia and astrocytic marker GFAP. Both lines exhibited GFAP expression at both time points. On day 52 GFAP+ cells appeared in patches throughout the organoid resembling radial glia, whereas on day 110 GFAP+ cells where mostly present in the outer rim of the organoid (Figure 1G). Apart from the neuroectodermal lineage markers, we stained control and D-CAA organoids for microglia using IBA1 at day 52 and day 110. Even though Lancaster and colleagues reported in their published protocol that more migratory cell types spread out of the organoid after Matrigel embedding, Quadrato et al. (2017) showed that microglia can develop in cerebral organoids. Moreover Ormel et al. (2018) showed that both with the protocol by Lancaster and colleagues, but also with a modified version, microglia innately developed in cerebral organoids. In our study, microglia were present in all cell lines examined, in approximately half of the sections (Figure 1H; Supplementary Figure 5). Overall, both control and D-CAA organoids display typical cortical region characteristics and express NPC-, deep layer neuron-and mature neuron-specific markers (Supplementary Figure 4).

### Targeted gene expression analysis of cell-type specific genes

To further examine specific cell types in a more sensitive and quantitative manner, the expression of cell type specific genes in whole D-CAA and control cerebral organoids from day 52 and day 110 was investigated. We assessed the expression of neuronal and glial genes using the Fluidigm qRT-PCR gene expression analysis platform.

**Figure 2:**
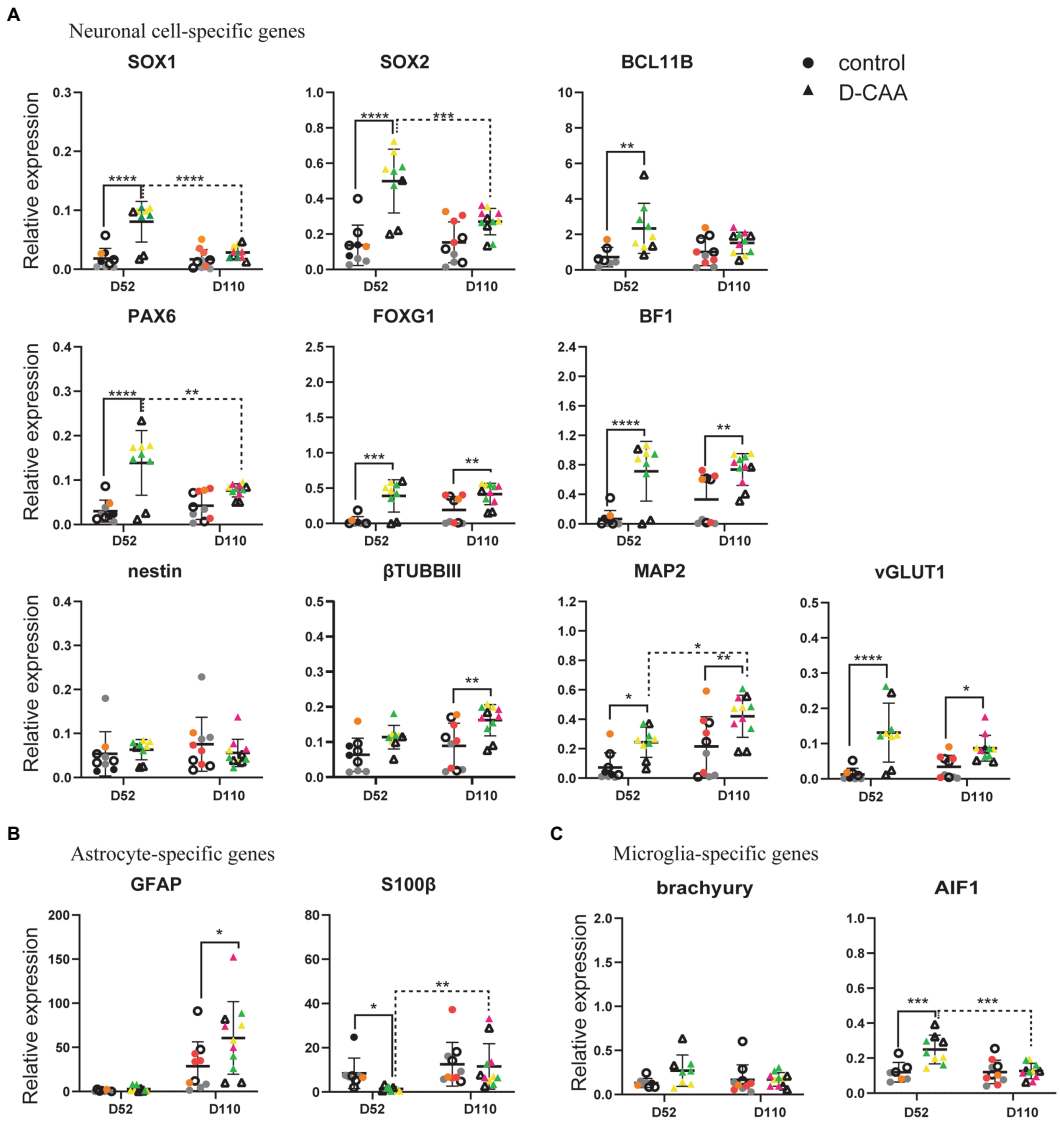
Targeted gene expression analysis of cell-type specific genes. **(A)** Enhanced neuronal differentiation and maturation in D-CAA cerebral organoids. **(B)** Assessment of microglia-and **(C)** astrocyte-specific genes. For every gene 4 control and 4 D-CAA lines were tested. Control and isogenic **D**-CAA lines are depicted with open circles and triangles, respectively. Three organoids per cell line were tested and for each gene technical duplicates were measured (± standard deviation (SD); *p ≤ 0.05, ****p ≤ 0.0001, two-way ANOVA with Bonferroni’s multiple comparisons test). Colored symbols represent replicates of individual cell lines.

To investigate the neuronal identity of cerebral organoids, we looked at NPC-specific genes, neuron-specific genes and forebrain neuron genes (Figure 2A). *SOX1* serves as the earliest marker for neural fate decision in stem cells and is also expressed in adult NPCs, while *SOX2* is expressed in stem cells and adult NSCs of the subventricular zone (SVZ) and is critical for NSCs proliferation and differentiation (Hsieh, 2012). *BCL11B* (also known as CTIP2) is a transcription factor that controls sub-cerebral projections from layer V neurons (Cánovas et al., 2015). Expression levels of *SOX1*, *SOX2*, and *BCL11B* were significantly higher in day 52 D-CAA organoids compared to controls, whereas at day 110 this difference was no longer significant. Furthermore, *SOX1* and *SOX2* levels in D-CAA lines significantly decreased from day 52 to day 110. Similarly *PAX6* expression was significantly elevated in D-CAA cerebral organoids compared to controls at day 52. This was no longer significant at day 110 due to a decrease of *PAX6* expression levels in the D-CAA lines. In line with a higher expression of *PAX6* in the D-CAA organoids, forebrain neuron-specific genes *BF1* and *FOXG1* also showed a significant increase in D-CAA cerebral organoids both at day 52 and 110. *Nestin* is an NPC marker but also a marker of neural stem cell (NSC) survival and self-renewal. *Nestin* showed similar expression levels between D-CAA and control organoids at both time points. *βTUBBIII* is a tubulin particularly involved in differentiation of NPCs and therefore considered a neuronal marker. *βTUBBIII* showed a higher expression in D-CAA lines at both time points but only reached significance at 110 days. In a similar fashion the dendrite-specific gene *MAP2* showed a significant higher expression in the D-CAA organoids, at both time points, indicating increased number of neurons. In the present study we did not perform any electrophysiology experiments to assess the neuronal functionality, however we looked at the gene expression levels of genes that are considered markers of mature neurons. We tested the organoids for expression of the vesicular glutamate transporters *vGLUT1* and *vGLUT2*, as well as the vesicular GABAergic transporter *vGAT*. *vGLUT1* and *vGLUT2* are concentrated in the synaptic vesicle; *vGLUT1* is identified in cerebral and cerebellar cortices and hippocampus whereas *vGLUT2* is mostly in diencephalon and rhombencephalon neurons (Yakoub and Sadek, 2018). Only *vGLUT1* expression increased significantly in D-CAA organoids compared to controls both at day 52 and day 110, while the other maturity markers were similar between the groups (Supplementary Figure 6).

Next, we assessed glial cell gene expression (Figure 2B; Supplementary Figure 6). The expression levels of the astrocyte relevant gene *GFAP* showed a significant increase at day 110 D-CAA organoids compared to controls as well as over time. Apart from *GFAP*, we examined expression of another astrocyte relevant gene namely *S100β*. In the study by Raponi et al. (2007), it was shown that *S100β* expression characterizes a critical period in which *GFAP*-expressing cells acquire a more mature developmental stage and lose their neural stem cell potential. *S100β* exhibited a significant decrease in D52 D-CAA organoids that returned to similar levels of control organoids at day 110. *S100β* is also considered to be an astrocytic marker of injury (Raponi et al., 2007). This suggests that in D-CAA organoids astrocytes mature later than control organoids. *OLIG2* is an oligodendrocyte-specific gene that showed no significant differences between control and D-CAA organoids (Supplementary Figure 6). To study changes in expression levels of several microglial genes we analyzed expression of *brachyury*, *AIF1 (IBA1)*, *P2RY12* and *TMEM119*. *AIF1* showed a significant increase in expression levels in day 52 D-CAA organoids compared to controls that was no longer significant at day 110. *Brachyury*, *P2RY12*, and *TMEM119* showed similar expression levels between the two conditions and time points (Figure 2C; Supplementary Figure 6).

Apart from comparing control and D-CAA cerebral organoids within a time point, we also looked at the changes within a condition across time. Overall our targeted gene expression results suggest an increased neuronal differentiation and maturation potential, enhanced forebrain neuronal development, as well as increased astrocytic and microglial gene expression in D-CAA organoids in comparison to controls.

### Targeted gene expression analysis in D-CAA organoids reflects deregulated genes found in D-CAA brain tissue

**Figure 3:**
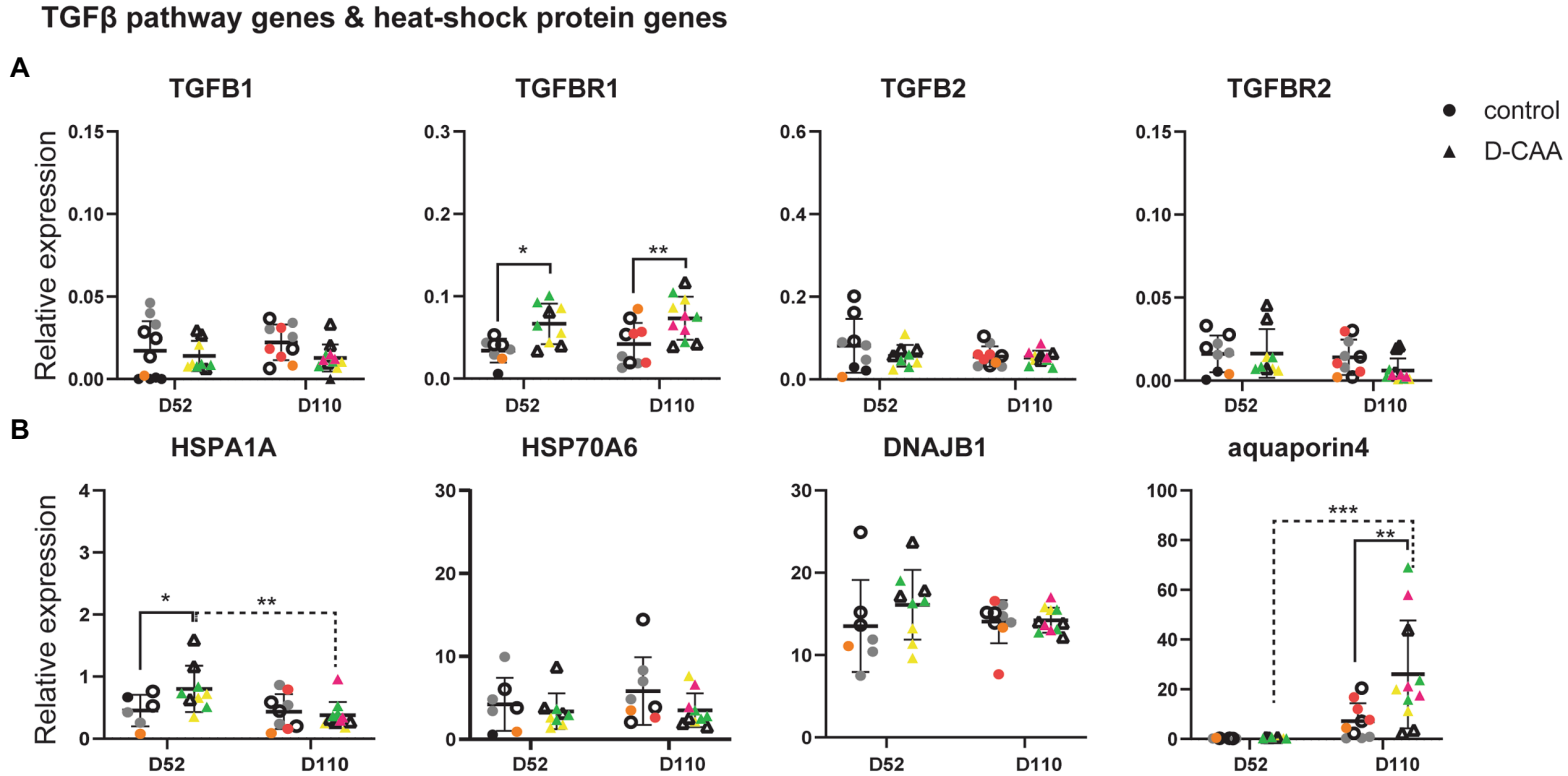
Targeted gene expression analysis of D-CAA relevant genes. **(A)** TGFB pathway gene analysis and **(B)** Heat-shock protein and aquaporin 4 expression analysis. For every gene 4 control and 4 D-CAA lines were tested. Control and isogenic D-CAA lines are depicted with open circles and triangles, respectively. Three organoids per cell line were tested and for each gene technical duplicates were measured (± SD; * p ≤ 0.05, **** p ≤ 0.0001, two-way ANOVA with Bonferroni’s multiple comparisons test). Colored symbols represent replicates of individual cell lines.

Next, we investigated gene expression changes known to be associated with D-CAA. Specifically, a previous study by our group in human D-CAA post-mortem brain tissue revealed an upregulation of the TGFβ pathway in D-CAA as well as an upregulation of several heat shock proteins (HSPs; Grand Moursel et al., 2018a,b). Transforming growth factor 1 (TGFβ1) plays a key role in vascular fibrosis by inducing extracellular matrix (ECM) proteins. Several *in vitro* studies have shown that TGFβ1 promotes APP and Aβ expression in astrocytes (Amara et al., 1999; Burton et al., 2002). The SMAD-dependent TGFβ pathway is activated upon binding of TGFB1 or TGFB2 to TGFB receptor 2 (TGFBR2), followed by phosphorylation of SMAD2 and 3 by TGFB receptor 1 (TGFBR1). In post-mortem brain tissue of D-CAA patients, TGFB1 and TGFBR2 genes were significantly increased compared to controls. Signaling genes of the canonical SMAD pathway (SMAD2, SMAD3, SMAD4, and SMAD7) were also assessed for gene expression changes but no significant difference was found (Grand Moursel et al., 2018a). In D-CAA organoids, the gene expression levels of TGFβ1, TGFβ2 and TGFBR2 did not show any significant differences compared to controls, while TGFBR1 displayed a significant increase in day 52 and day 110 D-CAA organoids (Figure 3A).

Our previous brain transcriptomic study of D-CAA post-mortem brain tissue also showed among other pathways, an upregulation of several heat shock protein family genes. HSPs are molecular chaperones important for handling and breakdown of misfolded proteins. We assessed the expression levels of *HSPA1A*, *HSPA6* and *DNAJB1* in cerebral organoids (Figure 3B). *HSPA1A* expression levels were significantly increased in day 52 D-CAA organoids but this difference was no longer significant at day 110. *HSPA6* and *DNAJB1* expression levels were similar between the two conditions and time points. Next we examined aquaporin 4 (*AQP4*), the main water-channel in mammalian brain that is expressed in astrocytes. A recent immunocytochemistry study in sCAA post-mortem human brain showed that AQP4 was significantly increased in moderate CAA compared to non-demented aging population significantly decreased in severe CAA (Owasil et al., 2020). In D-CAA organoids, a significant increase in *AQP4* expression levels was noticed at day 110 (Figure 3B). *AQP4* increase over time was similar to the increase in GFAP expression levels mentioned previously. Our findings from the targeted gene expression analysis confirm that even though cerebral organoids reflect better the embryonic brain, gene expression changes that are relevant to disease pathology found in human post-mortem brain tissue are present.

### D-CAA cerebral organoids exhibit Aβ accumulations

**Figure 4:**
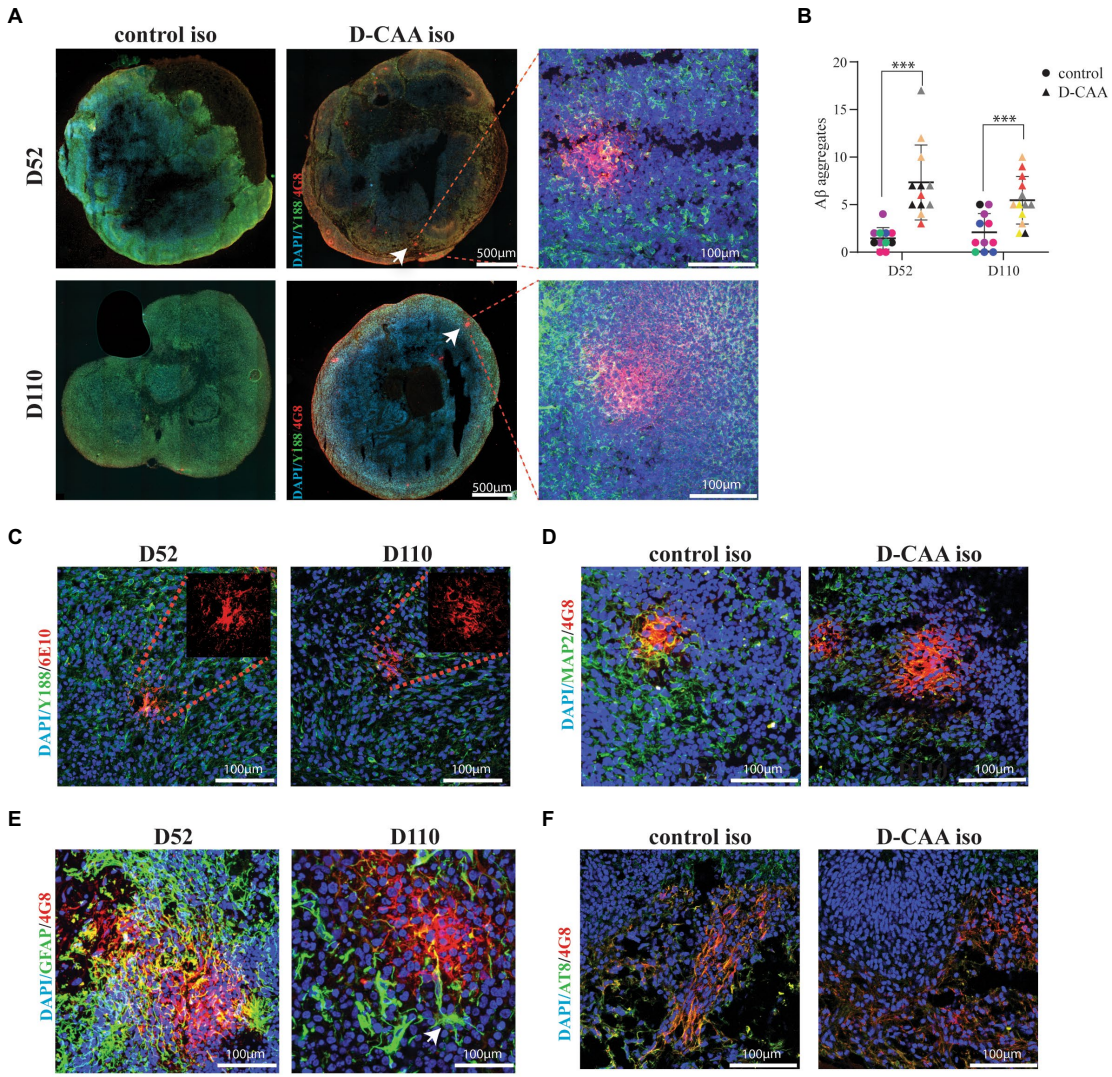
Aβ pathology in D-CAA cerebral organoids. **(A)** Representative immunofluorescent images of the isogenic control and D-CAA cerebral organoid lines at day 52 and 110 showing Aβ accumulations using the 4G8 antibody against Aβ (red) and the Y188 antibody against full-length APP (green). The white arrows point to the Aβ accumulations with a zoom in Confocal image to the right showing the Aβ accumulation at higher magnification. **(B)** Quantification of Aβ accumulations. For every cell line 3 organoids (biological triplicates) and 4 sections per organoid were checked for the presence of amyloid accumulations. **(C)** Validation of the 4G8 positive Aβ accumulations with a second anti-Aβ antibody, 6E10 (red). **(D)** Confocal images of a day 52 and day 110 D-CAA cerebral organoid section showing an Aβ accumulation (4G8-red) and MAP2 positive neurons (green). **(E)** Confocal images of a day 52 and day 110 D-CAA cerebral organoid section showing Aβ accumulation (4G8-red) and GFAP positive astrocytes (green), showing altered astrocyte morphology is altered in day 110 organoids (white arrow). **(F)** Confocal images of day 110 control and D-CAA isogenic pair cerebral organoids showing 4G8 Aβ positive cells (green) and phosphorylated tau positive cells (AT8-red).

A total of 188 forebrain D-CAA (107 sections) and control (81 sections) organoid sections from days 52 and 110 were immunofluorescently stained with antibodies against Aβ (4G8) and full length APP (Y188). In the D-CAA cell lines, Aβ accumulations were usually present in multiple sections per organoid (Figure 4A; Supplementary Figure 7). Quantification of the 4G8-specific Aβ accumulations of all D-CAA and control lines showed a significantly higher number of Aβ accumulations in the D-CAA organoids compared to controls at both time points (Figure 4B). To confirm that these accumulations were indeed Aβ we used a second Aβ-specific antibody (6E10) that recognizes the same Aβ accumulations (Figure 4C). To evaluate if there was a change in morphology of neurons in proximity of the Aβ accumulations, the D-CAAiso organoids of day 52 and day 110 were stained with the anti-Aβ antibody 4G8 and the neuronal antibody MAP2. We found no evidence of a change in neuronal morphology in close proximity of Aβ accumulations compared to neurons away from the accumulations (Figure 4D). Next we assessed the presence of microglia and astrocytes in close proximity to the Aβ accumulations in the D-CAAiso line. We did not find any IBA1 positive cells in close proximity to the Aβ accumulations. However, GFAP positive cells were present around every Aβ accumulation, both at day 52 and 110 (Supplementary Figure 8A). On both time points, the GFAP positive cells showed a change in morphology with more processes, that in some cases extended into the Αβ accumulation (Figure 4E; Supplementary Figure 9). In control D52 and D110 organoids, as well as in D-CAA D52 and D110 organoids but away from Aβ accumulations, GFAP positive cells showed mono-and bipolar morphologies (Supplementary Figure 9). Αβ aggregation has been already demonstrated in cerebral organoids carrying mutations in the presenilin and/or APP genes or overexpressing APP. Since these models study AD-related mutations, there is also tau pathology present (Murakami et al., 2003; Raja et al., 2016; Papaspyropoulos et al., 2020). Finally, we also looked at p-Tau expression in D-CAAiso and control organoid sections. We found no significant difference in p-Tau immunoreactivity between control and D-CAA organoids, a result that is also in line with D-CAA human brain tissue where no tau pathology present (Figure 4F; Supplementary Figure 8B).

## Discussion

In this study we present an *in vitro* cerebral organoid model that successfully recapitulates neurodevelopmental aspects of D-CAA pathology. D-CAA cerebral organoids exhibited Aβ accumulations, showed enhanced neuronal and astrocytic gene expression, as well as TGFβR1 upregulation in targeted gene expression analysis, compared to controls.

Cerebral organoids were generated from four control and four D-CAA hiPSC lines and were both qualitatively and quantitatively assessed for the expression of cell-type specific markers. In the hiPSC lines set, an isogenic D-CAA line generated with CRISPR/Cas9 using as template a control line was included. Creating an iPSC isogenic pair enables the assessment of molecular and cellular phenotypes that result in the present study from the Dutch mutation, without having any genetic background differences from the two cell lines. In the qualitative studies for Aβ pathology the D-CAA isogenic line was similar to the other D-CAA lines and distinct from its control counterpart. In the quantitative study though, that was not always the case. The D-CAA isogenic line showed variation between biological triplicates, however the same was seen in the rest of the organoid cell lines. That difference could be attributed to organoid-to-organoid variability and shows that more isogenic lines are needed for these experiments.

There are some considerations to take into account when using cerebral organoids as *in vitro* cell models, such as good reproducibility of the cerebral organoid protocol and low organoid-to-organoid variability. So far many studies have shown the successful generation of cerebral organoids that express a variety of cell types; however the variability between organoids, regarding size or morphology is not often shown (Lancaster et al., 2013, 2017; Qian et al., 2016; Gabriel and Gopalakrishnan, 2017; Quadrato et al., 2017). A recent study compared self-patterned and dorsally-patterned cerebral organoids protocols, using different cell lines and independent batches. They have shown that self-patterned organoids are highly variable in shape and external morphology compared to dorsally patterned organoids. When comparing single-cell RNA sequencing of 9 dorsally-patterned organoids from two cell lines with previously published datasets from self-patterned cerebral organoids and human fetal cerebral cortex, they showed that transcriptomic signatures from both organoid protocols highly correlated with human cells, however self-patterned cerebral organoids showed more organoid-to-organoid variability (Velasco et al., 2019). In our study we compared two different self-patterned cerebral organoid protocols to determine which protocol had the lowest variability regarding size and morphology. With the chosen protocol, eight cell lines were used to generate cerebral organoids. We showed that all cell lines displayed the same morphology in the most important stages of the cerebral organoid protocol and that throughout the culture time they maintained similar size. Moreover, immunofluorescent analysis with cell-type specific markers showed that these markers are expressed in all cell lines in a similar staining pattern. The targeted gene expression analysis that we performed for cell type specific markers also confirmed that the different control and D-CAA lines exhibit similar expression levels of the genes tested within each condition. Neuronal gene expression revealed that even though control and D-CAA cerebral organoids have similar neural stem cell potential and proliferation, the latter have elevated differentiation properties and lead to more forebrain neurons. In day 52 D-CAA organoids, neuronal precursor genes *SOX1*, *SOX2* and *PAX6*, were significantly increased in D-CAA compared to controls. In line with the neuronal precursors, forebrain neuron genes *FOXG1* and *BF1*, as well as *MAP2* and the mature neuronal marker *VGLUT1*, were also significantly increased. These results suggest that there might be changes already in early brain development, even though disease onset is in mid-life. These results complement the differences seen by immunofluorescent analysis in cortical plate marker expression in control organoids where less organized cortical plates were visualized at day 52 compared to D-CAA organoids. Such early changes have also be suggested for other adult onset neurodegenerative disorders such as Huntington disease (HD), where it was shown that there are early neurodevelopmental defects leading to premature neuronal differentiation (Zhang et al., 2018). Regarding D-CAA, APP itself could have an effect on the enhanced neuronal differentiation and maturation, as it has been showed by multiple studies to have an effect in neurogenesis, neuronal proliferation and division, as it is highly expressed during brain development (Löffler and Huber, 1992; Hayashi et al., 1994; Ohsawa et al., 1999; Zhang et al., 2014). A study on hiPSC neurons and brain organoids carrying the Swedish mutation (causing familial AD) showed enhanced neuronal maturation and aberrant electrical activity in comparison with isogenic controls (Ghatak et al., 2019). Another D-CAA signature revealed by an RT-qPCR quantitative study of D-CAA and control post-mortem brain tissue was the upregulation of the TGFβ signaling pathway that was also later confirmed as an upregulated pathway by an RNA-sequencing study (Grand Moursel et al., 2018a,b). The transcriptomic study has also shown up-regulation of genes from the heat-shock protein (HSP) family (Grand Moursel et al., 2018b). We only observed upregulation of *TGFBR1* whereas the rest of the TGFβ pathway genes remained unchanged. Gene expression analysis of the human fetal brain has shown that *TGB1*, *TGFBR1*, and *TGFBR2* are expressed early, in post-conception week 4, in a similar expression pattern with *PAX6* and *nestin*, and decrease over time (Izsak et al., 2020). A study in human iPSC-NSC cultures has shown that TGFβ genes and their ligands are expressed in these cell populations and that TGFβ1 acts as a positive regulator and promotes neuronal differentiation of NSC cultures, as well as promotes the appearance of immature GFAP+ cells in early NSC cultures. Using 3D cultures they noticed that TGFβ1 treatment led to increased number of neurons with an increasing, but not significant tendency to functionality. They concluded that TGFβ1 is not the only signaling cue (Izsak et al., 2020). In the present study, *TGFBR1* increased expression in D-CAA organoids follows the increased expression pattern of the NPC markers *SOX1, SOX2, PAX6*. Moreover, *TGFBR1* has been found to be abundantly expressed in the neuronal cells of the rat cerebral cortex (Tomoda et al., 1996; Pál et al., 2014) and that is also true for the D-CAA cerebral organoids that exhibit simultaneous increased expression of the cortical neuronal genes *FOXG1* and *BF1*. The differences in the D-CAA pathology observed between the post-mortem brain tissue and cerebral organoids could be attributed to the fact that cerebral organoids represent the prenatal brain and consider the upregulation as an early disease event. Furthermore, the significant increase of HSPA1A expression at day 52 suggests that D-CAA organoids could be responding to the Aβ accumulating peptide.

The Dutch mutation has been studied widely for its aggregation properties, where it was found that the E22 amino acid change to Q22 indeed creates a peptide that is more prone to form aggregates (Melchor et al., 2000; Van Nostrand et al., 2001; Murakami et al., 2003; Baumketner et al., 2008). The main pathological finding in the brains of D-CAA patients is accumulation of mainly the Aβ40 peptide around blood vessels, however the mechanism behind this perivascular aggregation is still poorly understood. Together with a less efficient clearance of Aβ from the brain, there is higher concentration of Aβ around the blood vessels, favoring Aβ aggregation. Here, we showed Aβ accumulations in cerebral organoids of endogenous APP/Aβ expression with a consistent significant increase of Aβ accumulations in organoids from D-CAA patients compared to controls in both day 52 and day 110 time points. This could be due to the tightly packed cells of the organoid where diffusion of proteins and peptides is reduced, causing locally higher concentrations that would favor aggregation.

In D-CAA patients there is no tau pathology, which is in line with our findings in D-CAA cerebral organoids. *In vitro* and *in vivo* AD research have shown that there are morphological differences in various cell types around Aβ plaques such as neuronal loss around Aβ aggregates and activated astrocytes and microglia (González-Reyes et al., 2017; Navarro et al., 2018). In the D-CAA organoids, no morphological changes were seen in neurons and microglia in close proximity to the Aβ accumulation compared to the ones further away from the accumulation, which that suggests these Aβ accumulations represent a very early and immature disease feature. However we noticed that in 90% of Aβ accumulations astrocytes surrounded the accumulation and showed long extensions protruding the Aβ accumulation. Astrocytic involvement in D-CAA was also evident from the targeted gene expression analysis that revealed a significant increase in astrocytic markers at day 110 D-CAA organoids compared to controls and a dramatic increase across time. Apart from *GFAP*, *AQP4* that is the main water-channel expressed in astrocytes and a marker of CAA pathology was significantly increased, confirming an astrocytic pathology in D-CAA organoids. An additional astrocytic marker, *S100β* present in a variety of studies that have shown its expression in cerebral organoids was also assessed (Yakoub, 2019; Logan et al., 2020; Sivitilli et al., 2020) and showed a decreasing trend in day 52 D-CAA organoids. Mouse studies revealed that S100B is expressed at a late developmental stage after which GFAP-expressing cells lose their NSC potential and acquire an astrocytic phenotype. (Raponi et al., 2007). Based on this evidence we therefore hypothesize that astrocytes in D-CAA cerebral organoids mature later than controls, a result that could be attributed to Aβ pathology in these organoids. The lack of vasculature in cerebral organoids and the low-throughput are general limitations for brain organoids and this D-CAA organoid model. Recently a research group has successfully created bioengineered vessels to study the progress and molecular mechanisms that lead to cerebrovascular amyloid angiopathy after addition of amyloid beta fibrils to the vessels (Shin et al., 2019). An *in vitro* BBB model was also constructed from iPSC-derived vascular cells for AD (Blanchard et al., 2020). However, these models lack the neuronal part of the brain. More complex models that include all the different brain cell entities are needed to reach higher compatibility with the human brain.

## Conclusion

In this study, enhanced neuronal differentiation, Aβ accumulations and TGFβ pathway de-regulation were found in organoids of D-CAA patients. Cerebral organoids hold great potential for *in vitro* disease modeling and with thorough characterization they will be able to serve as suitable platforms for unraveling molecular mechanisms of various diseases as well as therapeutic intervention testing, such as antisense oligonucleotide (AON) therapies. Treating D-CAA cerebral organoids with AONs would be the next step to discover if these compounds are able to reverse the pathology that was found in the current D-CAA organoid model. Although the current D-CAA organoid model is limited by the lack of vasculature and shows more resemblance to the early stages of brain development, our results show that this *in vitro* model provides a promising tool that can help with therapy development for D-CAA and other Aβ accumulation disorders.

## Data availability statement

The original contributions presented in the study are included in the article/Supplementary material, further inquiries can be directed to the corresponding authors.

## Author contributions

ED conceived the study, performed the experiments, did the analysis, and wrote the manuscript. BP performed the CRISPR/Cas9 experiments. SS performed part of the experiments. LG provided technical knowledge and support. GT provided the patient skin biopsies. DP and RB provided supervision of the methodology. WR-M conceived and supervised the study. All authors contributed to the article and approved the submitted version.

## Funding

The authors declare that this study received funding from Amylon Therapeutics B.V. The funder was not involved in the study design, collection, analysis, interpretation of data, the writing of this article or the decision to submit it for publication.

## Conflict of interest

The authors declare that the research was conducted in the absence of any commercial or financial relationships that could be construed as a potential conflict of interest.

## Publisher’s note

All claims expressed in this article are solely those of the authors and do not necessarily represent those of their affiliated organizations, or those of the publisher, the editors and the reviewers. Any product that may be evaluated in this article, or claim that may be made by its manufacturer, is not guaranteed or endorsed by the publisher.

## Supplementary material

SUPPLEMENTARY FIGURE S1 Quality controls of D-CAA iPSC lines, **(A)** Brightfield images of D-CAA iPSC colonies revealing iPSC morphology with tightly packed cells, **(B)** Pluripotency test of D-CAA iPSCs with immunofluorescent stainings using antibodies against SSEA-4, Nanog and Oct3/4, **(C)** Pluripotency gene expression analysis for NANOG, OCT4 and SOX2 genes, **(D)** Global screening array (GSA) showed no evidence of copy number variants or allelic changes in 2 D-CAA lines, **(E)** Sanger sequencing confirmation of the Dutch mutation (G>C), **(F)** Immunofluorescent analysis of D-CAA iPSC spontaneous differentiation to the three germ layers shown with antibodies against SOX17 (endoderm), SMA (mesoderm) and PAX6 (ectoderm).

SUPPLEMENTARY FIGURE S2 Generation of an isogenic D-CAA line with CRISPR/Cas9 technology (D-CAAiso). **(A)** Brightfield images of 2 D-CAA iPSC colonies revealing iPSC morphology with tightly packed cells, **(B)** Sanger sequencing confirmation of the Dutch mutation (G>C), **(C)** Immunofluorescent analysis of D-CAAiso spontaneous differentiation to the three germ layers shown with antibodies against SOX17 (endoderm), SMA (mesoderm) and PAX6 (ectoderm).

SUPPLEMENTARY FIGURE S3 Comparison of two cerebral organoid protocols; Stemcell Technologies cerebral organoid differentiation kit and protocol by protocol described by Gabriel et al. 2017 [30]. **(A)** Brightfield images of control 1 and D-CAA 3 cell lines. The images depict the following steps of the cerebral organoid protocol; EB formation (day 5), neuronal rosette expansion (day 10), and cerebral organoid maturation (day 20). **(B)** Size quantification of control 1 and D-CAA 3 organoids for the first 36 days in culture. For every time point 2-6 images for every organoid were taken, **(C)** Immunofluorescent analysis of D52 and D110 control organoid section with antibodies against PAX6 (green) and βTUBBIII (red). Nuclei are stained with DAPI.

SUPPLEMENTARY FIGURE S4 Cortical plate formation in D-CAA and control organoids. Immunofluorescent analysis of cortical plates from D52 and D110 control and D-CAA organoids using antibodies against PAX6 (green), CTIP2 (yellow) and βTUBBIII (red). Nuclei are stained with DAPI.

SUPPLEMENTARY FIGURE S5 Microglia are present in all control and D-CAA organoids. Immunofluorescent analysis of D52 control and D-CAA organoids with an antibody against IBA1 (red). Nuclei are stained with DAPI.

SUPPLEMENTARY FIGURE S6 Targeted gene expression analysis of neuronal-, astrocytic- and microglia-specific genes.

SUPPLEMENTARY FIGURE S7 Aβ accumulation in D52 and D110 control and D-CAA cerebral organoids. Immunofluorescent analysis with antibodies against Aβ (4G8-red) and fl-APP (Y188-green). White arrows indicate Aβ accumulations.

SUPPLEMENTARY FIGURE S8 **(A)** Astrocytes are surrounding Aβ accumulations. Immunofluorescent analysis of D52 and D110 D-CAA organoid sections with an anti Aβ antibody (4G8-red) and an anti-GFAP antibody marking astrocytes, **(B)** Quantification of pTau immunoreactivity in five control and D-CAA section. pTau immunoreactivity was normalized against MAP2 or control.

SUPPLEMENTARY FIGURE S9 Astrocyte morphology around the Aβ accumulations. Immunofluorescent analysis of D52 and D110 control and D-CAA organoid sections with antibodies against Aβ (4G8-red) and GFAP (green). Nuclei are stained with DAPI. **(A)** GFAP+ astrocytes in control D52 and D110 organoids; white circles indicate selected astrocytes that show mono-or bi- polar morphologies, **(B)** GFAP+ astrocytes away or in close proximity to the Aβ accumulation; white circles indicate selected organoids that show either monopolar/bipolar or multiple processes respectively.
