# Supplementary figures and images for: Amyloid beta accumulations and enhanced neuronal differentiation in cerebral organoids of Dutch-type cerebral amyloid angiopathy patients
Source: Front Aging Neurosci. 2023 Jan 17;14:1048584. doi: 10.3389/fnagi.2022.1048584 (PMC9887998; doi:10.3389/fnagi.2022.1048584)

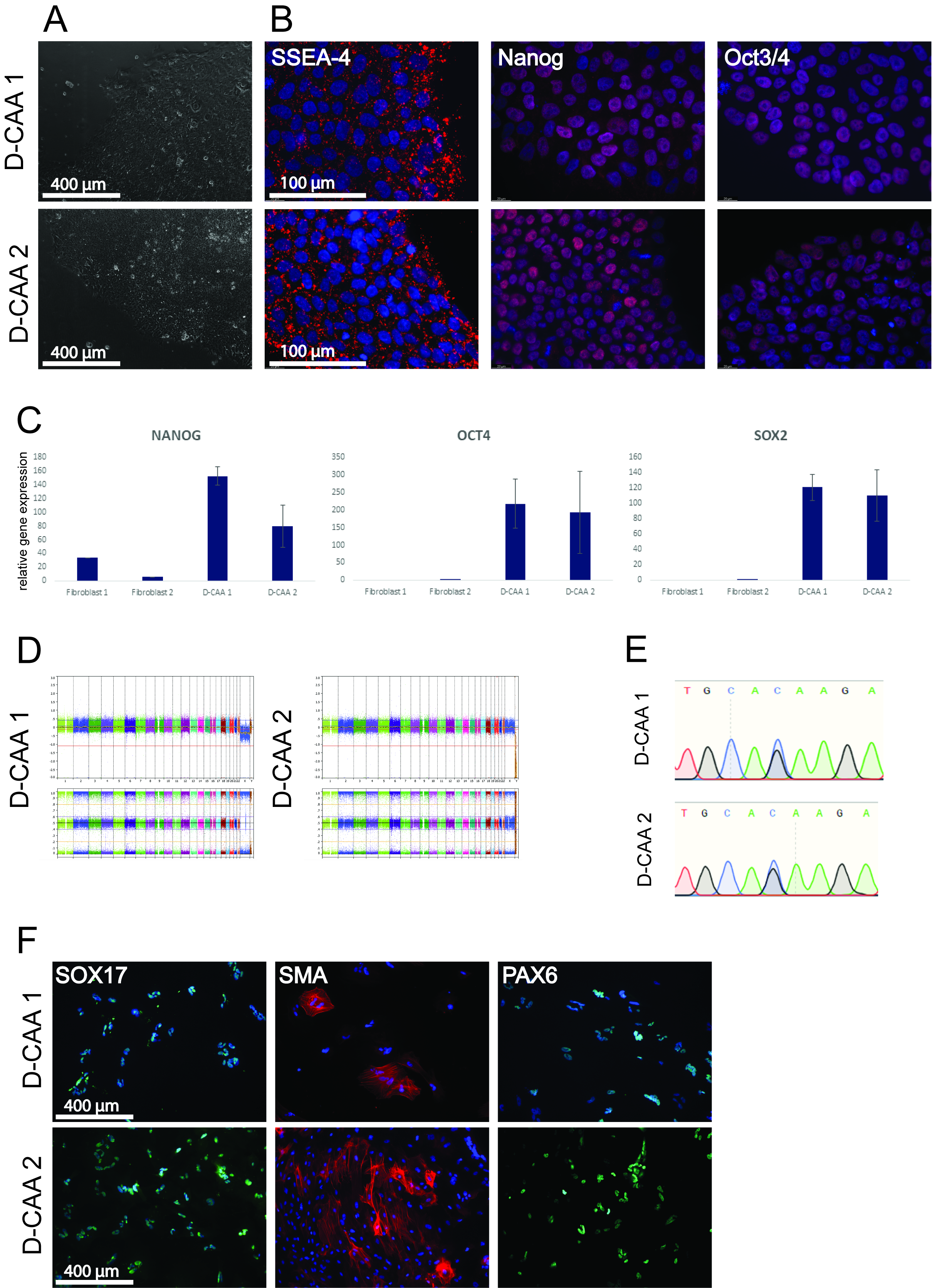

Supplement: SUPPLEMENTARY FIGURE S1 — Quality controls of D-CAA iPSC lines, (A) Brightfield images of D-CAA iPSC colonies revealing iPSC morphology with tightly packed cells, (B) Pluripotency test of D-CAA iPSCs with immunofluorescent stainings using antibodies against SSEA-4, Nanog and Oct3/4, (C) Pluripotency gene expression analysis for NANOG, OCT4 and SOX2 genes, (D) Global screening array (GSA) showed no evidence of copy number variants or allelic changes in 2 D-CAA lines, (E) Sanger sequencing confirmation of the Dutch mutation (G>C), (F) Immunofluorescent analysis of D-CAA iPSC spontaneous differentiation to the three germ layers shown with antibodies against SOX17 (endoderm), SMA (mesoderm) and PAX6 (ectoderm). [file Image_1.TIF]

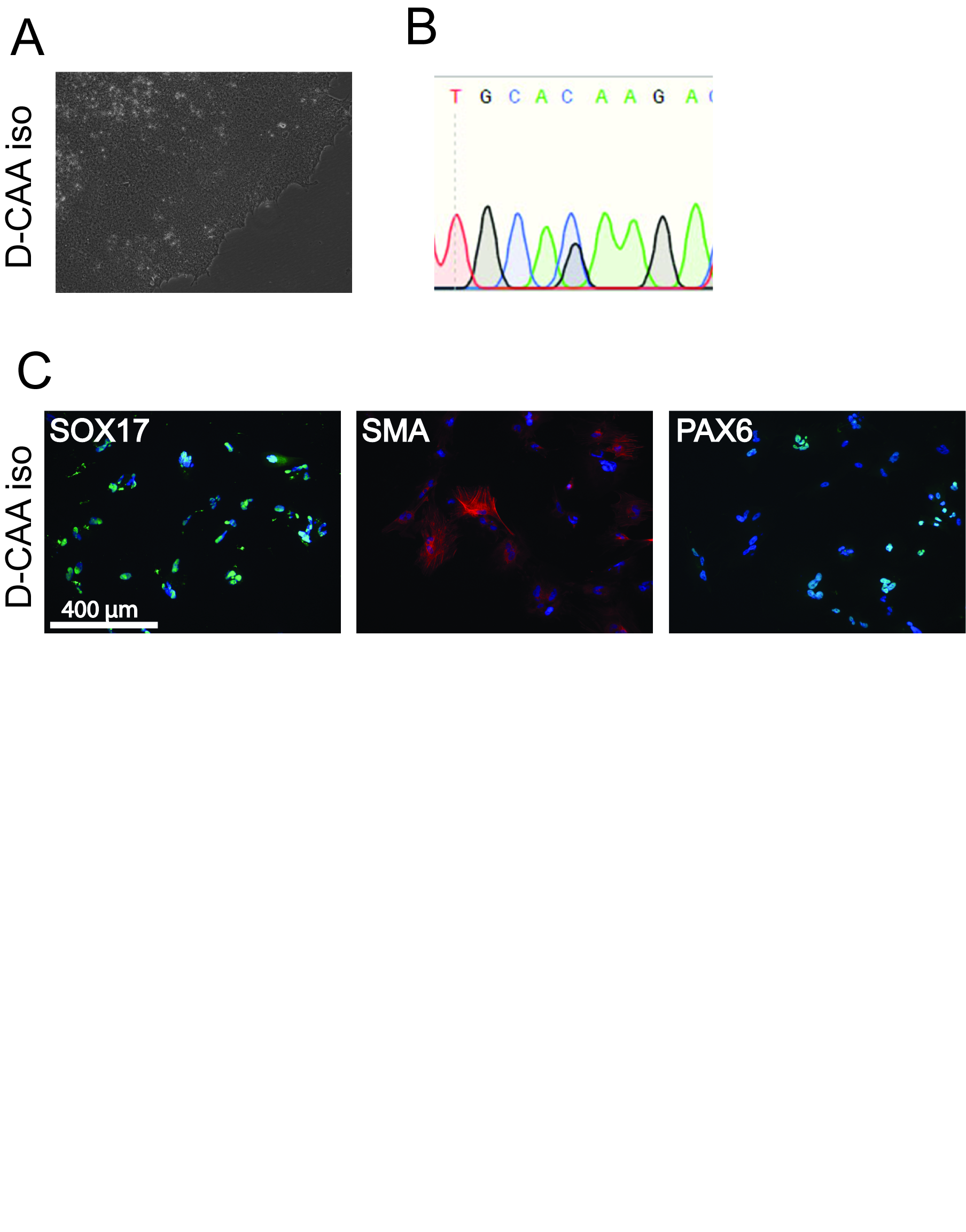

Supplement: SUPPLEMENTARY FIGURE S2 — Generation of an isogenic D-CAA line with CRISPR/Cas9 technology (D-CAAiso). (A) Brightfield images of 2 D-CAA iPSC colonies revealing iPSC morphology with tightly packed cells, (B) Sanger sequencing confirmation of the Dutch mutation (G>C), (C) Immunofluorescent analysis of D-CAAiso spontaneous differentiation to the three germ layers shown with antibodies against SOX17 (endoderm), SMA (mesoderm) and PAX6 (ectoderm). [file Image_2.TIF]

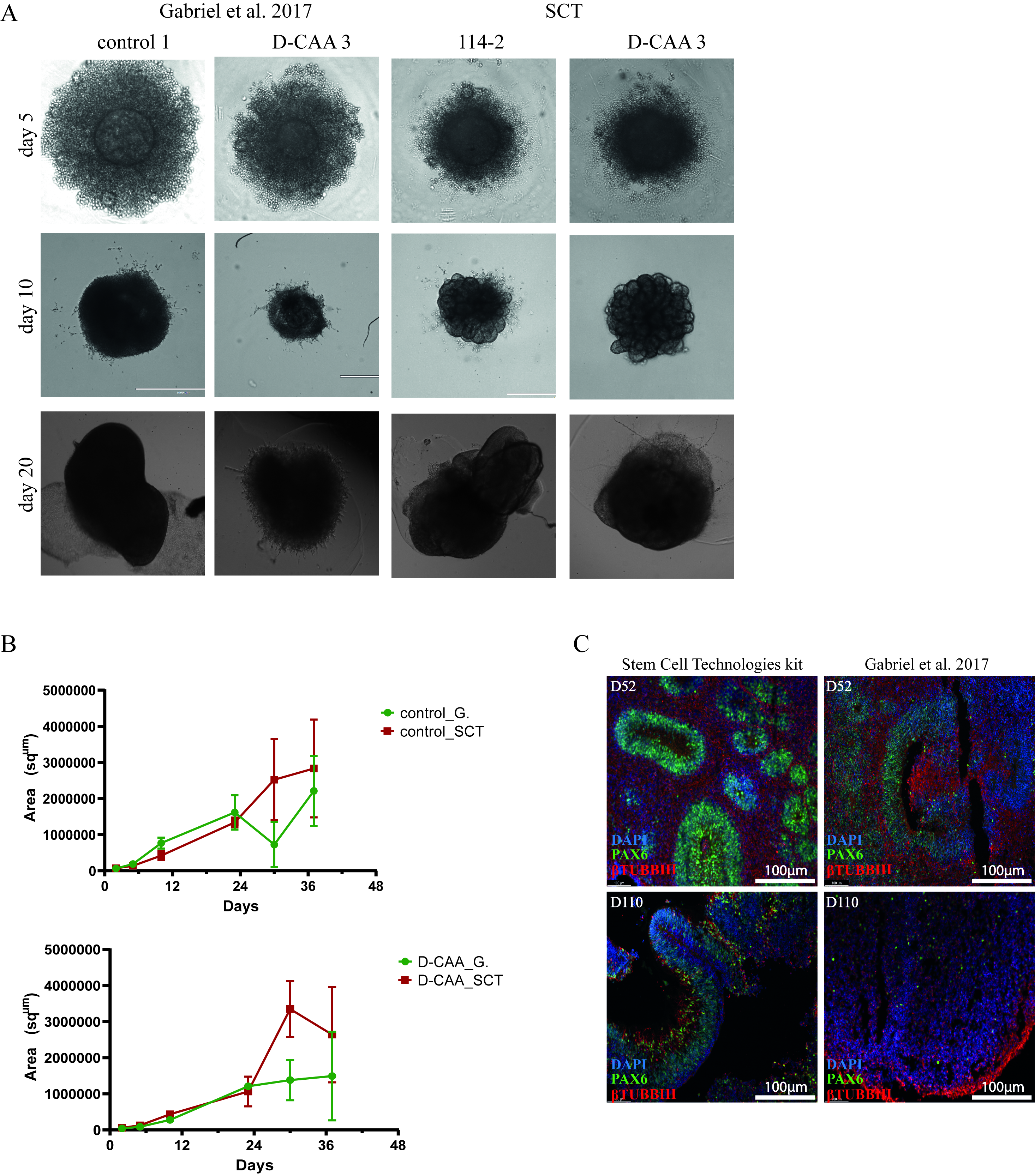

Supplement: SUPPLEMENTARY FIGURE S3 — Comparison of two cerebral organoid protocols; Stemcell Technologies cerebral organoid differentiation kit and protocol by protocol described by Gabriel et al. 2017 [30]. (A) Brightfield images of control 1 and D-CAA 3 cell lines. The images depict the following steps of the cerebral organoid protocol; EB formation (day 5), neuronal rosette expansion (day 10), and cerebral organoid maturation (day 20). (B) Size quantification of control 1 and D-CAA 3 organoids for the first 36 days in culture. For every time point 2-6 images for every organoid were taken, (C) Immunofluorescent analysis of D52 and D110 control organoid section with antibodies against PAX6 (green) and βTUBBIII (red). Nuclei are stained with DAPI. [file Image_3.TIF]

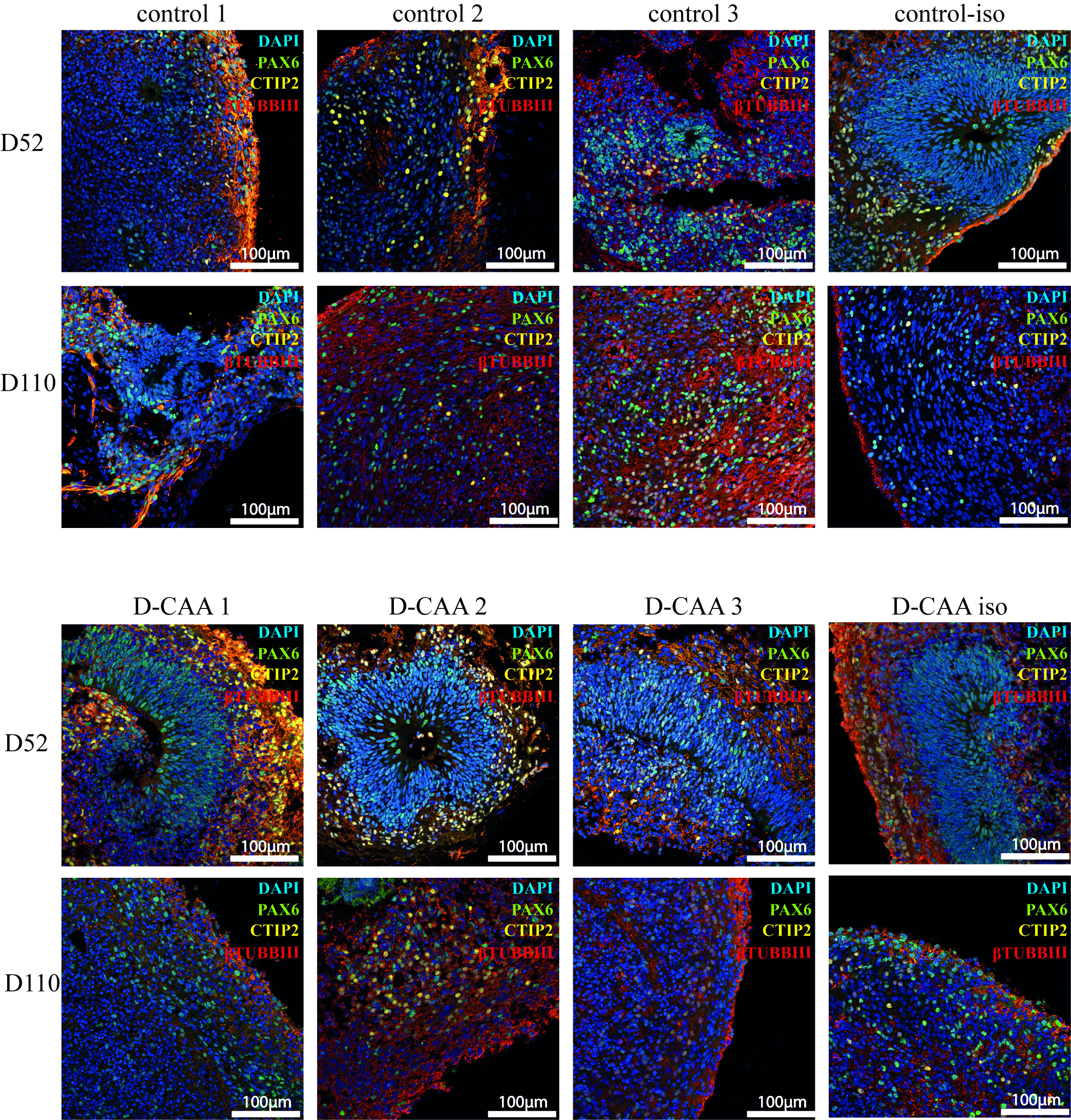

Supplement: SUPPLEMENTARY FIGURE S4 — Cortical plate formation in D-CAA and control organoids. Immunofluorescent analysis of cortical plates from D52 and D110 control and D-CAA organoids using antibodies against PAX6 (green), CTIP2 (yellow) and βTUBBIII (red). Nuclei are stained with DAPI. [file Image_4.TIF]

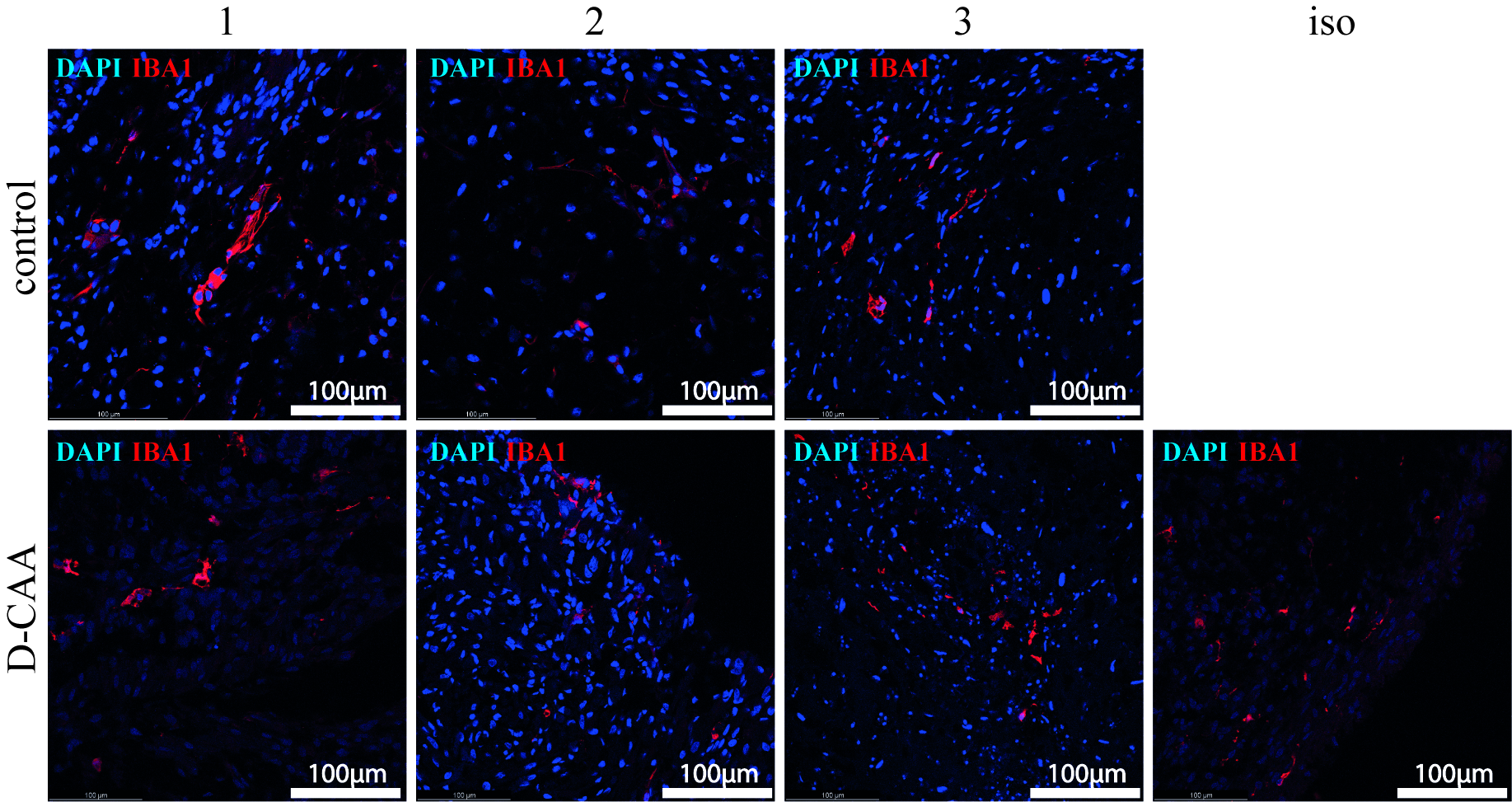

Supplement: SUPPLEMENTARY FIGURE S5 — Microglia are present in all control and D-CAA organoids. Immunofluorescent analysis of D52 control and D-CAA organoids with an antibody against IBA1 (red). Nuclei are stained with DAPI. [file Image_5.TIF]

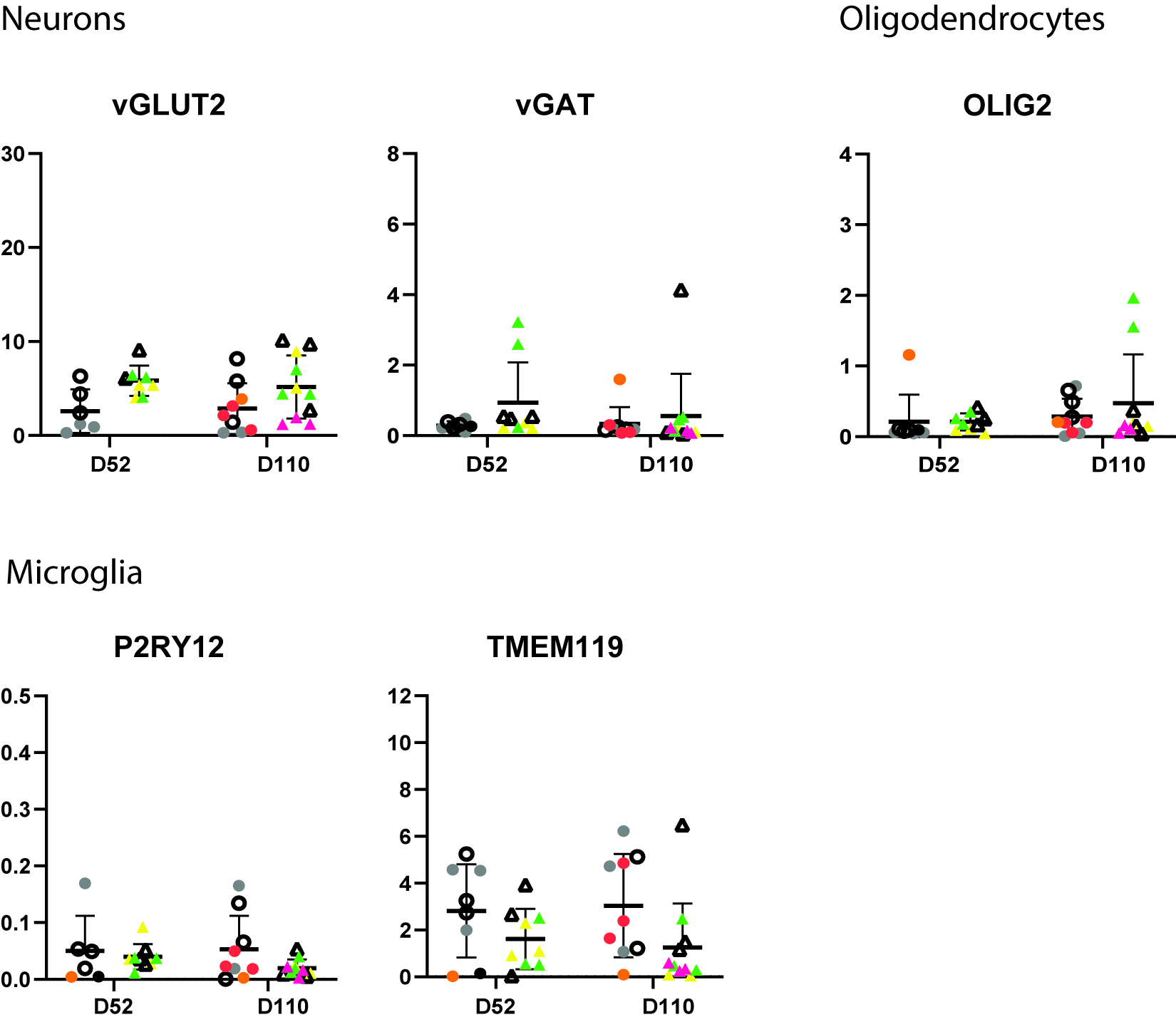

Supplement: SUPPLEMENTARY FIGURE S6 — Targeted gene expression analysis of neuronal-, astrocytic- and microglia-specific genes. [file Image_6.TIF]

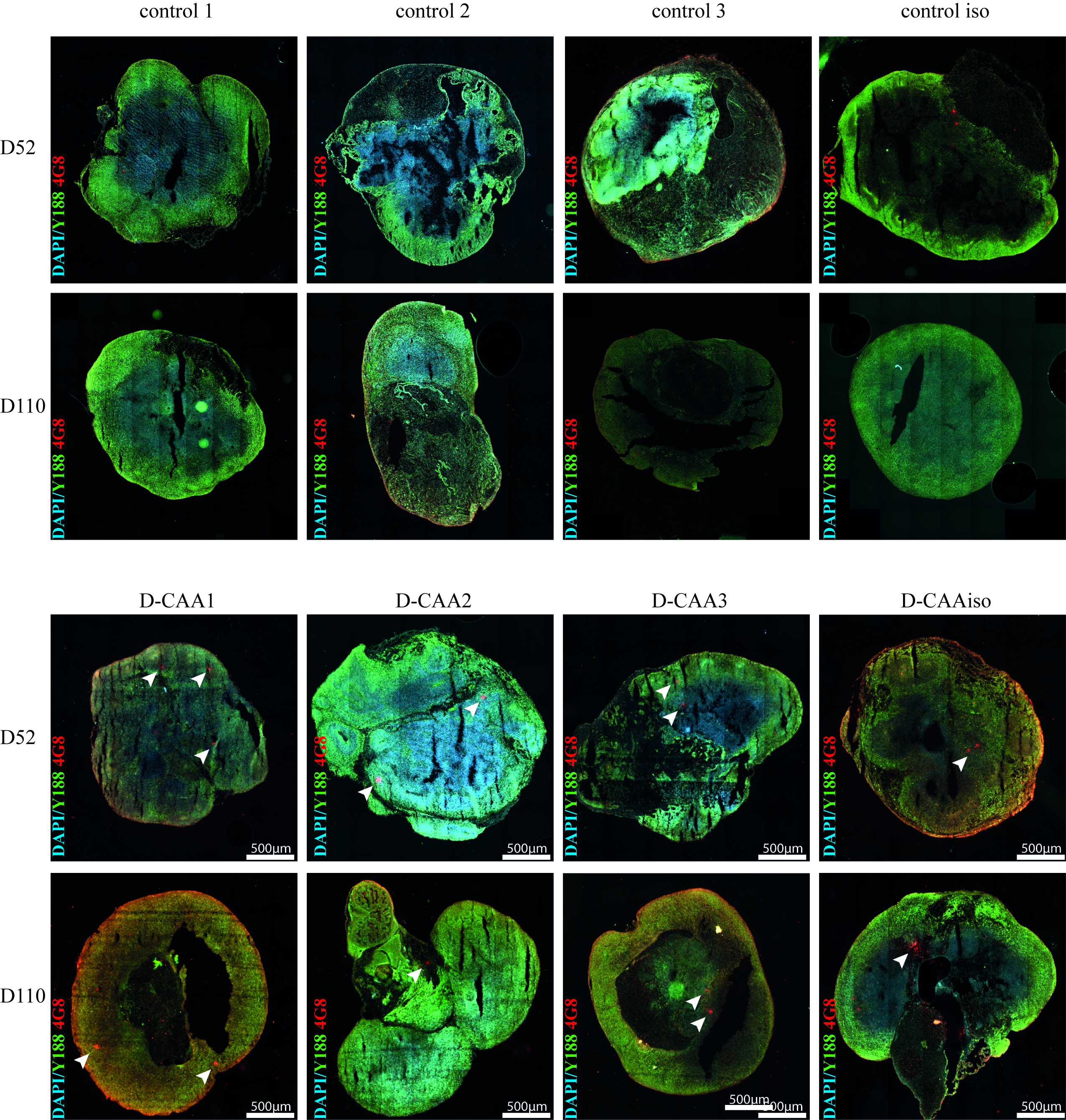

Supplement: SUPPLEMENTARY FIGURE S7 — Aβ accumulation in D52 and D110 control and D-CAA cerebral organoids. Immunofluorescent analysis with antibodies against Aβ (4G8-red) and fl-APP (Y188-green). White arrows indicate Aβ accumulations. [file Image_7.tif]

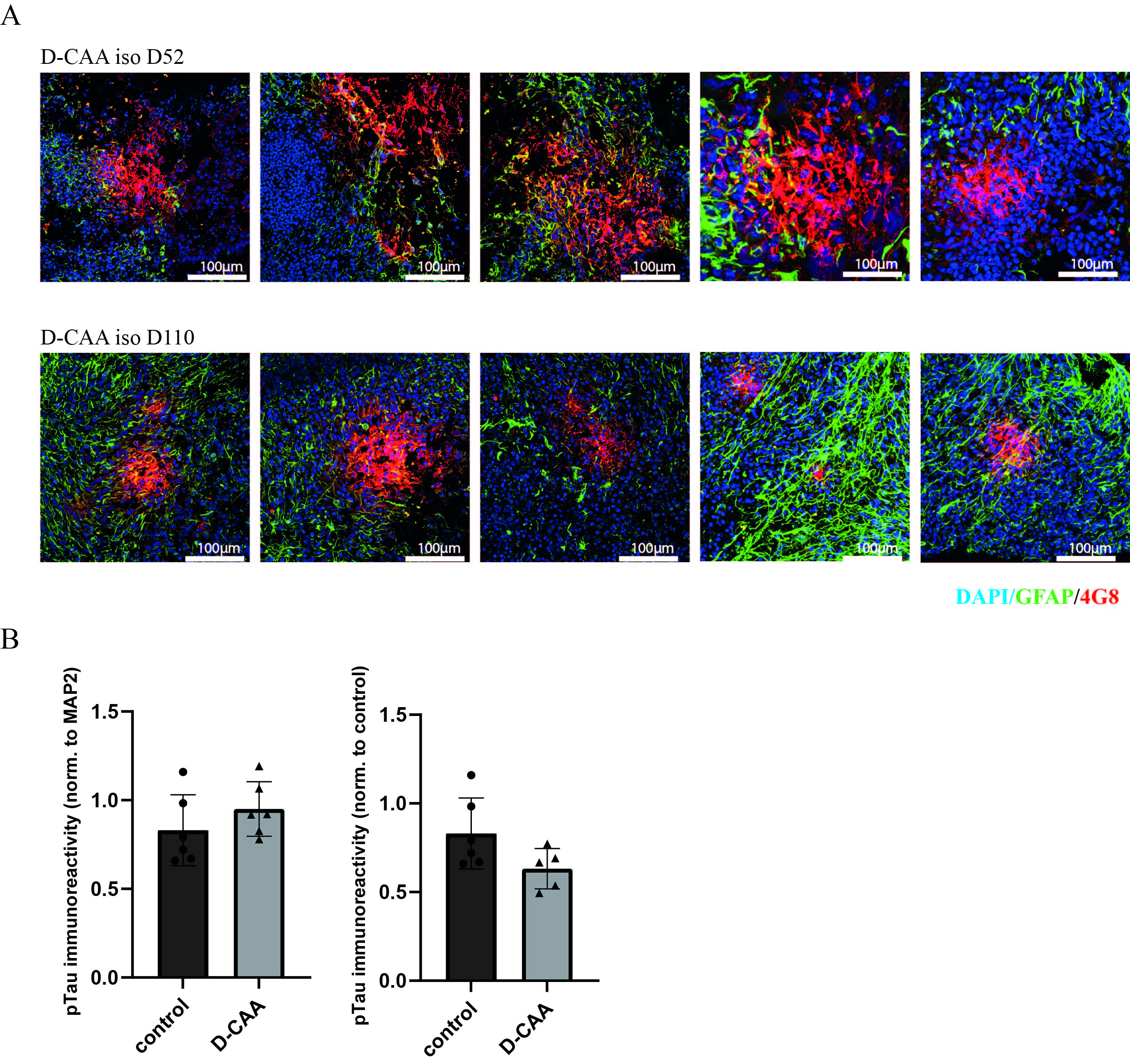

Supplement: SUPPLEMENTARY FIGURE S8 — (A) Astrocytes are surrounding Aβ accumulations. Immunofluorescent analysis of D52 and D110 D-CAA organoid sections with an anti Aβ antibody (4G8-red) and an anti-GFAP antibody marking astrocytes, (B) Quantification of pTau immunoreactivity in five control and D-CAA section. pTau immunoreactivity was normalized against MAP2 or control. [file Image_8.jpg]

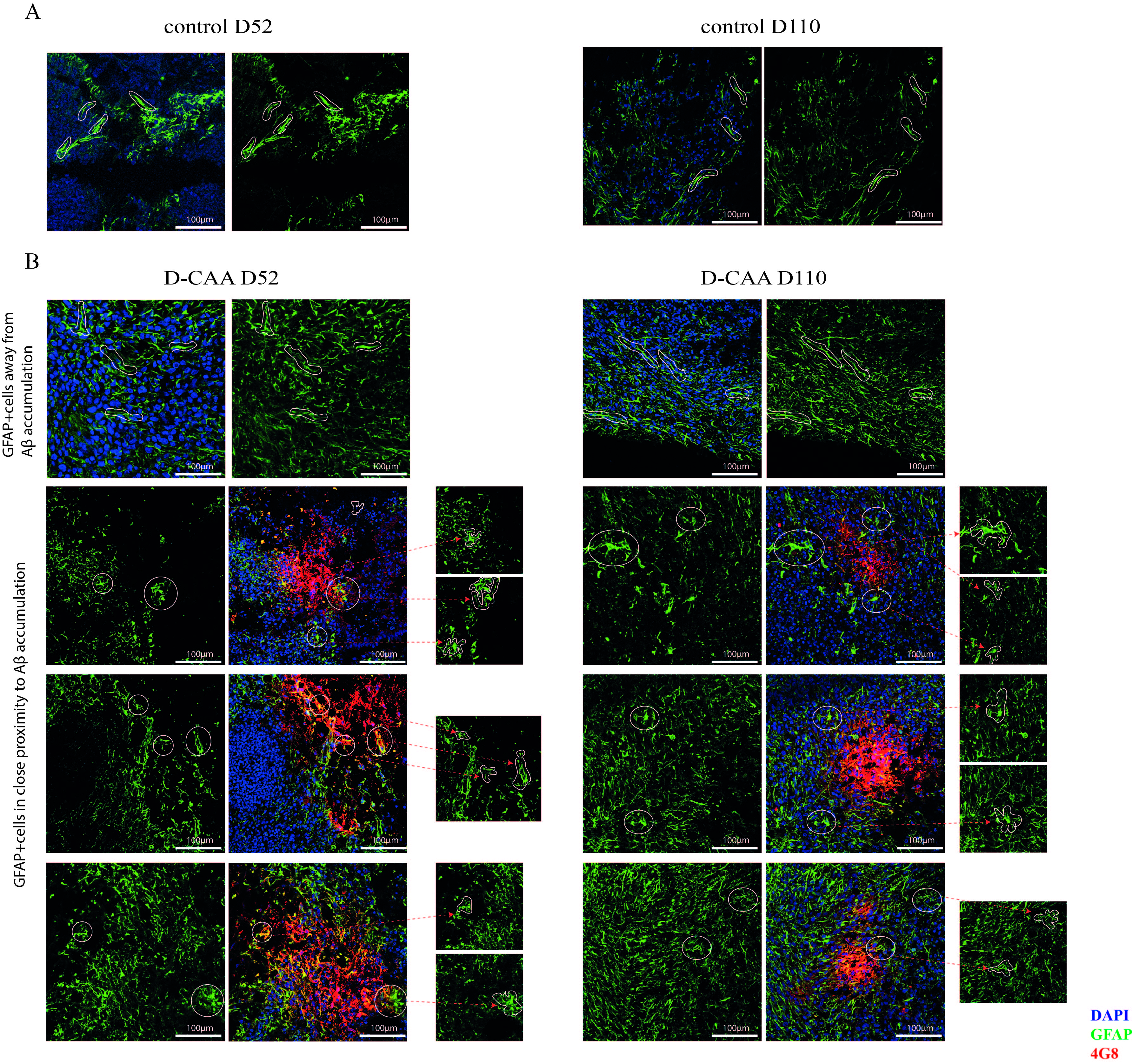

Supplement: SUPPLEMENTARY FIGURE S9 — Astrocyte morphology around the Aβ accumulations. Immunofluorescent analysis of D52 and D110 control and D-CAA organoid sections with antibodies against Aβ (4G8-red) and GFAP (green). Nuclei are stained with DAPI. (A) GFAP+ astrocytes in control D52 and D110 organoids; white circles indicate selected astrocytes that show mono-or bi- polar morphologies, (B) GFAP+ astrocytes away or in close proximity to the Aβ accumulation; white circles indicate selected organoids that show either monopolar/bipolar or multiple processes respectively. [file Image_9.jpg]
